# Synthesis and Biological Evaluation of Lipophilic 1,4-Naphthoquinone Derivatives against Human Cancer Cell Lines

**DOI:** 10.3390/molecules200711994

**Published:** 2015-06-30

**Authors:** Shao-Hung Wang, Chih-Yu Lo, Zhong-Heng Gwo, Hong-Jhih Lin, Lih-Geeng Chen, Cheng-Deng Kuo, Jin-Yi Wu

**Affiliations:** 1 Department of Microbiology, Immunology and Biopharmaceuticals, College of Life Sciences, National Chiayi University, Chiayi 60004, Taiwan; E-Mails: shwang@mail.ncyu.edu.tw (S.-H.W.); s0990613@mail.ncyu.edu.tw (Z.-H.G.); s1030730@mail.ncyu.edu.tw (H.-J.L.); lgchen@mail.ncyu.edu.tw (L.-G.C.); 2 Department of Food Science, College of Life Sciences, National Chiayi University, Chiayi 60004, Taiwan; E-Mail: chihyulo@mail.ncyu.edu.tw; 3 Department of Medical Research and Education, Taipei Veterans General Hospital, Taipei 11217, Taiwan; E-Mail: cdkuo23@gmail.com

**Keywords:** 1,4-naphthoquinone, terpenoids, anticancer activity, human colon cancer cells HT-29, cell cycle distribution, apoptosis

## Abstract

To examine the effect of hydrophobicity on the anticancer activity of 1,4-naphthoquinone derivatives, a series of compounds bearing a 2-*O*-alkyl-, 3-*C*-alkyl- or 2/3-*N*-morpholinoalkyl group were synthesized and evaluated for their anticancer activity against five human cancer cell lines *in vitro*. The cytotoxicity of these derivatives was assayed against HT-29, SW480, HepG2, MCF-7 and HL-60 cells by the MTT assay. Among them, 2-hydroxy-3-farnesyl-1,4-naphthoquinone (**11a**) was found to be the most cytotoxic against these cell lines. Our results showed that the effectiveness of compound **11a** may be attributed to its suppression of the survival of HT-29. Secondly, in the Hoechst 33258 staining test, compound **11a**-treated cells exhibited nuclear condensation typical of apoptosis. Additionally, cell cycle analysis by flow cytometry indicated that compound **11a** arrested HT-29 cells in the S phase. Furthermore, cell death detected by Annexin V-FITC/propidium iodide staining showed that compound **11a** efficiently induced apoptosis of HT-29 in a concentration-dependent manner. Taken together, compound **11a** effectively inhibits colon cancer cell proliferation and may be a potent anticancer agent.

## 1. Introduction

**Figure 1:**
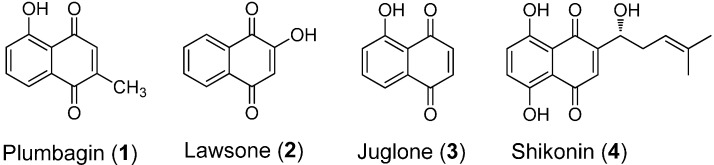
Chemical structures of plumbagin (**1**); lawsone (**2**); juglone (**3**) and shikonin (**4**).

Millions of lives are lost because of cancer in the world each year and the number is increasing. However, greater than 30% of cancers are preventable [1] and some forms of cancer are curable regardless of when they are diagnosed, while many others are only curable if discovered at an early stage. Thus cancer remains a life-threatening disease and represents a serious peril to human health [2]. Chemotherapy is vital to cancer treatment and the majority of clinical antitumor agents have originated from natural products. Plants produce a wide range of secondary metabolites such as flavonoids, terpenoids, alkaloids, quinones, polyacetylene, and sugar as part of their defense mechanism against insects [3] and microbials [4]. Among these, 1,4-naphthoquinones, in particular, plumbagin (**1**), lawsone (**2**), juglone (**3**), and shikonin (**4**) [5] (Figure 1), are an important group of natural products, which have been employed in the development of potent phytochemicals because of their high abundance and relatively non-toxic nature. The 1,4-naphthoquinone pharmacophore is known to impart anticancer activity in a number of drugs, such as streptonigrin [6], mitomycins [7], and doxorubicin [8] among others. The anticancer activity of the 1,4-naphthoquinone skeleton has been the focus of recent research in the field [9,10].

Among 1,4-naphthoquinones, there are numerous interesting biologically active compounds such as atovaquone, plumbagin, lapachol, and menadione [11]. Nevertheless, a literature precedence revealed that plumbagin (5-hydroxy-2-methyl-1,4-naphthoquinone), a wide type quinonoid, found in the plants of the Plumbaginaceae, Droseraceae, Ancestrocladceae, and Dioncophyllaceae families, possesses significant antimicrobial [12], antimalarial [13], antifilarial [14], antiprotozoal [15], anti-inflammatory [16], and anticancer properties [17,18,19].

Further, it was reported that monoterpenyl- and diterpenylnaphthoquinones derivatives had IC_50_ values in the micromolar range against several tumor cell lines [20]. These derivatives were synthesized through Diels-Alder addition of natural terpenoids and *p*-benzoquinones, followed by side chain transformation [21]. Additionally, several 1,4-naphthoquinone derivatives incorporating with hydroxyl groups at C-5 can provide enhanced anti-proliferative activity in *in vivo* and *in vitro* [22].

It was also reported that 1,4-naphthoquinones interfere with the electron transport and oxidative phosphorylation processes and play roles in enzyme inhibition, and DNA cross linking as well as have antifungal, antibacterial, and anticancer activities [7]. Nonetheless, many reported amino-1,4-naphthoquinones did not contain substituents on the benzene ring, although some studies [21,22] have shown that derivatives with side chains (alkyl or alkenyl) on the benzene ring considerably improved the cytotoxicity of the 1,4-naphthoquinone moiety.

In this study, we synthesized and evaluated the effect of incorporation of new alkyl or terpenyl moieties in 1,4-naphthoquinones, including lawsone substituted at the 2-*C*/3-*O*-position with alkyl or terpenyl groups and juglone substituted at position 2/3-*N*-morpholinoalkyl groups, with the aim to analyze the influence of these substituents on the cytotoxicity of these derivatives against human cancer cell lines. We prepared and evaluated C-2 amino substituted 1,4-naphthoquinones, a core structure present in numerous naturally occurring bioactive quinones, such as mitomycin C, as well as some synthetic compounds [23].

## 2. Results and Discussion

### 2.1. Chemistry 

**Scheme 1:**
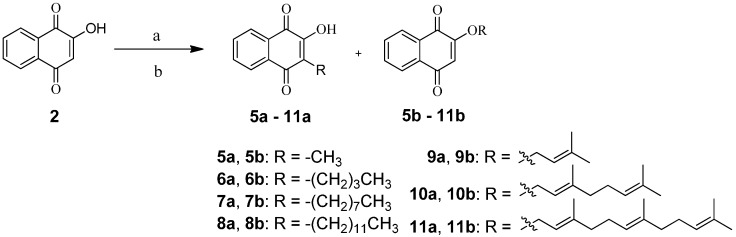
Synthesis of compounds **5a**,**b**‒**11a**,**b**.

In general, 1,4-naphthoquionone derivatives were synthesized from lawsone (**2**) with alkyl, terpenyl, alkylamino, or morpholinealkylamino moieties through 2-*O*- or 3-*C*-alkylation. As shown in Scheme 1, synthesis of **5a**,**b**–**11a**,**b** were carried out by reacting lawsone with selected alkyl or terpenyl bromide in dimethylformamide (DMF) using triethylamine as the base and sodium iodide (NaI) as the catalyst under N_2_ for 1 h according to the reported procedure [24]. The addition of an alkyl or terpenyl group to lawsone (**2**) gave two products at the 2-*O*-postion and 3-*C*-position in 40.0%–72.3% yield. The 3-*C*-substituted compounds were the major products while the 2-*O*-substituted compounds were obtained with a yield of less than 10%.

*Reagents and conditions*: (**a**) NaI, triethylamine, DMF, N_2_, 1 h; (**b**) alkyl or terpenyl bromide, 40 °C, 3–7 days.

**Scheme 2:**
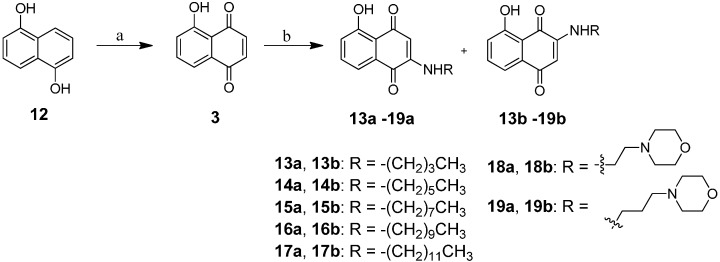
Synthesis of compounds **13a**,**b**‒**19a**,**b**.

The synthesis of juglone (**3**) was via oxidation of commercially available 1,5-dihydroxy-naphthalene (**12**) in air, and dark, in the presence of periodic acid with a yield of 67.6%. Chemical transformation of juglone (**3**) to compounds **13a**,**b**‒**19a**,**b** was then carried out according to the reported methods with some modifications [25]. Aminated juglone (**3**) was prepared by stirring it with a long chain amine in ethanol for 1 h under reflux to give 2-alkylaminojuglone and 3-alkylaminojuglone as major and minor products, respectively. Lastly, once the alkylamino substituted 1,4-naphthoquinone derivatives were obtained, other alkyl- or morpholinealkylamino-juglones were prepared by adding primary alkyl amines and morpholinealkyl amines to juglone (**3**) in ethanolic solution at room temperature. The two-step reaction and end products are illustrated in Scheme 2.

*Reagents and conditions*: (**a**) HIO_4_, THF/H_2_O, rt, 30 min; (**b**) alkyl amine, EtOH, reflux, 1–5 h.

### 2.2. In Vitro Anti-Proliferative Activity by MTT Assay

The cytotoxic effects of the 31 1,4-naphthoquinone derivatives were tested *in vitro* against five human cancer cell lines: HT29 (colorectal adenocarcinoma), SW480 (colorectal adenocarcinoma), HepG2 (hepatocellular carcinoma), HL60 (leukemia), MCF-7 (breast adenocarcinoma) and normal murine embryonic liver BNL CL.2 cells, using the 3-[4,5-dimethylthiazol-2-yl]-2,5-diphenyltetrazolium bromide (MTT) assay [26]. 5-Fluorouracil (5-Fu), cisplatin and doxorubicin were used as positive controls. The concentration (in μM) of the test compounds which induced 50% inhibition of cell growth (IC_50_) is shown in Table 1.

Of the 31 compounds tested, eleven compounds were bioactive and nine (**5b**, **6b**, **7b**, **10a**, **11a**, **18a**, **18b**, **19a** and **19b**) showed significant cell growth inhibition in four human cancer cells except MCF-7 cells. In addition, the anticancer activity of these compounds increased with the higher chain length of the terpenyl substituents, but not in long chain alkyl groups. This result suggests that the lipophilicity of terpenyl moiety of the 1,4-naphthoquinone derivatives at the 3*-C*-substituent affected the bioactivity of the compound (**11a**). In particular, among the bioactive ones, compound **11a** exhibited the highest activity against the human colorectal HT-29 cell lines with an IC_50_ value of 1.99 ± 0.04 μM for 48 h. Compared to plumbagin (**1**)’s IC_50_ value of 3.67 ± 0.46 μM, derivative **11a** was slightly more cytotoxic against human colorectal carcinoma HT-29 cells. No significant cell death was detected in normal murine embryonic liver BNL CL.2 cells treated with plumbagin (**1**) and **11a**. Furthermore, in BNL CL.2 cells, only a marked effect on cell death (under 20%) was observed at the maximum concentration (20 μM) of these two compounds after 48 h treatment (see also Figure S1). It thus appeared that both plumbagin (**1**) and compound **11a** are cytotoxic to human colorectal cancer cells with no significant adverse side effects toward normal cells. Namely, the lipophilic characteristics of the substituent at the 3-C-position of the farnesyl moieties of 1,4-naphthoquinone derivative **11a** can remarkably enhance their anticancer activity against human cancer HT-29 cell lines. In other words, a higher anticancer activity because the longer terpenyl moiety of the 1,4-naphthoquinone derivative **11a** enhanced their cell membrane permeability and bioavailability, thus increasing their cytotoxic activities.

**Table 1:** IC_50_ values of plumbagin (**1**), lawsone (**2**) and its derivatives **5a**,**b**–**11a**,**b**, **13a**,**b**‒**19a**,**b** on the growth of human cancer cell lines for 48 h.

Compound	cLog*P* ^a^	IC_50_ (μM) ^b^ (Mean ± SD)				
		HT-29 Colon	SW480 Colon	HepG2 Liver	HL60 Leukemia	MCF-7 Breast
Plumbagin (**1**)	2.78	3.67 ± 0.46	2.19 ± 0.65	2.32 ± 0.18	5.90 ± 1.21	20.89 ± 0.70
Lawsone (**2**)	1.68	>100	>100	>100	45.12 ± 7.90	>100
Juglone (**3**)	2.26	>50	>50	>50	46.95 ± 0.58	>50
**5a**	2.19	>100	97.09 ± 2.36	94.08 ± 2.63	>100	>100
**5b**	1.78	8.16 ± 1.16	4.23 ± 0.50	3.32 ± 0.66	16.40 ± 2.84	>25
**6a**	3.79	>100	>100	>100	>100	>100
**6b**	3.22	14.81 ± 0.65	2.02 ± 0.20	3.67 ± 0.84	20.04 ± 0.79	31.03 ± 0.32
**7a**	5.90	33.44 ± 1.48	9.73 ± 0.04	20.20 ± 3.52	>100	>50
**7b**	5.34	28.01 ± 3.52	8.51 ± 0.53	9.66 ± 0.50	23.42 ± 1.24	20.81 ± 3.67
**8a**	7.46	>25	>25	>25	>25	>25
**8b**	8.02	>25	>25	>25	>25	>25
**9a**	3.70	>100	>100	74.18 ± 2.61	>100	>100
**9b**	3.34	>50	20.72 ± 1.19	35.56 ± 1.13	>50	>50
**10a**	5.73	12.90 ± 2.13	14.14 ± 0.96	19.10 ± 0.26	9.12 ± 0.69	>100
**10b**	5.37	42.90 ± 1.13	44.23 ± 1.06	29.10 ± 1.21	29.56 ± 0.88	>100
**11a**	6.82	1.99 ± 0.04	2.02 ± 0.31	14.01 ± 2.70	5.41 ± 0.88	47.01 ± 0.47
**11b**	7.40	40.37 ± 0.75	37.16 ± 2.21	32.33 ± 1.46	35.10 ± 1.52	>100
**13a**	3.65	9.82 ± 1.51	16.17 ± 0.72	9.39 ± 0.12	45.69 ± 2.48	>100
**13b**	3.65	18.11 ± 1.68	8.28 ± 0.12	17.89 ± 0.14	6.50 ± 0.32	23.71 ± 0.82
**14a**	4.99	>50	>50	>50	>50	>50
**14b**	4.99	34.53 ± 1.20	15.13 ± 0.49	17.82 ± 2.29	>50	>50
**15a**	6.05	>50	>50	>50	>50	>50
**15b**	6.05	>50	20.23 ± 2.18	47.06 ± 0.63	>50	>50
**16a**	7.11	>50	>50	>50	>50	>50
**16b**	7.11	>50	>50	>50	>50	>50
**17a**	8.17	>20	>20	>20	>20	>20
**17b**	8.17	>20	>20	>20	>20	>20
**18a**	2.51	9.42 ± 0.45	8.86 ± 0.50	11.72 ± 0.42	8.42 ± 0.86	>50
**18b**	2.51	8.59 ± 0.67	5.19 ± 0.44	8.84 ± 0.07	6.49 ± 0.84	>50
**19a**	2.80	9.44 ± 0.32	4.66 ± 0.25	10.08 ± 0.57	6.35 ± 0.24	>50
**19b**	2.80	11.38 ± 1.51	3.86 ± 0.61	7.90 ± 0.16	7.76 ± 0.54	>50
Cisplatin	-	24.07 ± 0.03	40.72 ± 1.18	36.07 ± 3.11	>100	>100
5-Fu	-	>100	32.72 ± 8.32	40.18 ± 7.63	>100	>100
Doxorubicin	-	1.70 ± 0.20	0.53 ± 0.07	0.30 ± 0.02	14.26 ± 0.89	0.39 ± 0.06

^a^ Calculated log value of partition coefficient by ChemDraw Ultra 8.0; ^b^ IC_50_ = compound concentration required to inhibit tumor cell proliferation by 50%. Data are expressed as the mean ± SD from the dose response curves of at least three independent experiments.

### 2.3. Cytotoxic Activity of Plumbagin (**1**) and Compound **11a** was Exhibited in a Time-Dependent Manner

In light of the cytotoxicity findings described above, we extended our study on the anti-proliferation efficacy of plumbagin (**1**) and lawsone derivative **11a** against HT-29 cells to 24 h, 48 h and 72 h periods. The data on plumbagin (**1**) was used here as base case. As shown in Table 2, first of all, the cytotoxicity of compound **11a** demonstrated, once again stronger bioactivity than that of plumbagin (**1**) *in vitro*. In addition, the growth inhibition of HT-29 cells induced by these two samples was in a time-dependent manner. The IC_50_ values of plumbagin (**1**) and **11a** in HT-29 cells for 24 h, 48 h and 72 h incubation times were 4.72, 3.67, 2.10 μM and >20, 1.99, 0.84 μM, respectively. The results revealed that plumbagin (**1**) and **11a** inhibited the cell viability of HT-29 cells in a time-dependent manner at longer time.

**Table 2:** Cytotoxicity of plumbagin (**1**) and **11a** on HT-29 for 24 h, 48 h, and 72 h.

Compound	IC_50_ (μM) ^a^ (Mean ± SD)		
	24 h	48 h	72 h
Plumbagin (**1**)	4.72 ± 0.07	3.67 ± 0.46	2.10 ± 0.23
**11a**	>20	1.99 ± 0.04	0.84 ± 0.12

^a^ IC_50_ = compound concentration required to inhibit tumor cell proliferation by 50%. Data are expressed as the mean ± SD from the dose response curves of at least three independent experiments.

### 2.4. Nuclear Morphological Changes of HT-29 Cells Treated with Plumbagin (**1**) and Compound **11a**

**Figure 2:**
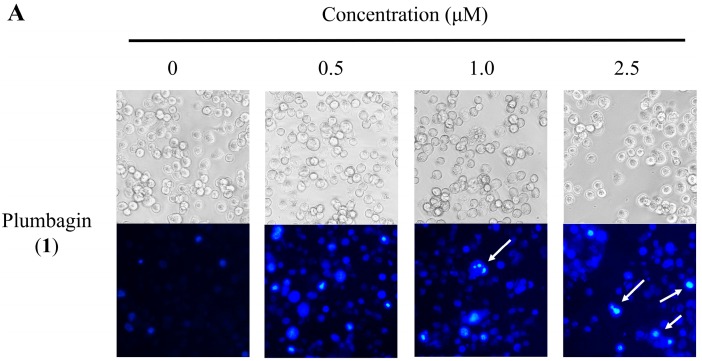

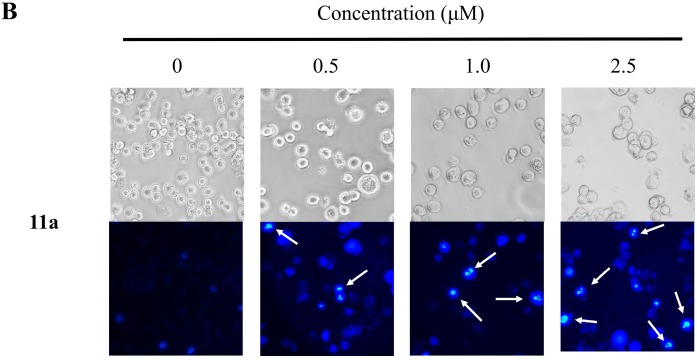
The morphological changes of HT-29 cells treated with 0–2.5 μM plumbagin (**1**) (**A**) and **11a** (**B**) for 48 h. (magnification, 200×). (**A**,**B**) The upper panels showed the cell morphology under phase-contrast microscopy, and the lower panels display the Hoechst 33258-stained nuclear patterns take by fluorescence microscopy (magnification, 200×).

Apoptosis, shown by morphological changes, were detected through Hoechst 33258 staining in the HT-29 cells. The classical characteristics of apoptotic cells are chromatin condensation and nuclear fragmentation [27]. To further investigate the role apoptosis plays in the superior cytotoxicity of plumbagin (**1**) and the potent compound **11a**, we incubated HT-29 cells, with plumbagin (**1**) and **11a**, separately, for 48 h. The cells were then stained with Hoechst 33258, and examined by fluorescence microscopy for topical morphological changes. As shown in Figure 2, while the nuclei of the cells were round in shape and stained homogenously in the control without testing compound, those treated with plumbagin (**1**) and **11a** showed typical morphological features of apoptotic cells based on cell shrinkage, chromatin condensation and nuclear fragmentation [28]. Evidently, the proliferation of the cancer cell is inhibited by the testing compounds *in vitro*.

### 2.5. Cell Cycle Distribution Analysis Using Flow Cytometry

**Figure 3:**
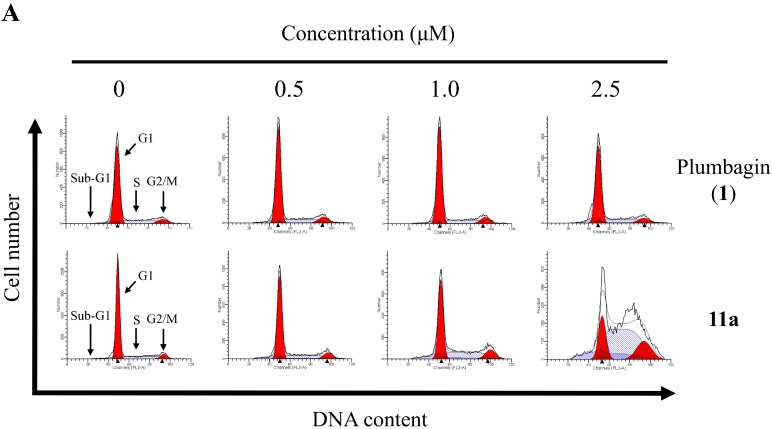

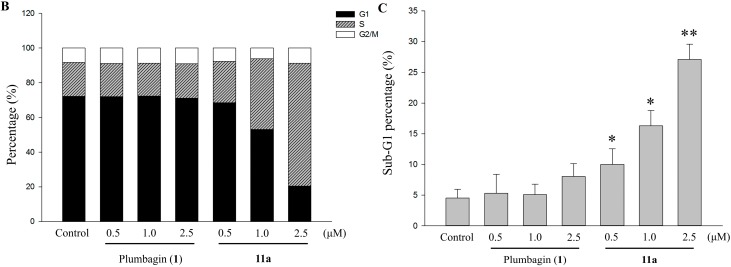
(**A**) The effects of the 48 h treatments with 0–2.5 μM plumbagin (**1**) and **11a** on cell cycle distribution of HT-29. After treatment, cells were fixed and stained with PI, and the cell cycle distribution was examined by flow cytometry; (**B**) Quantitative data of cell cycle analysis of HT-29 treated with 0‒2.5 μM plumbagin (**1**) and **11a** for 48 h; (**C**) Compared with control, the treated with plumbagin (**1**) and **11a** shows the obvious increase in sub-G1 fraction. Each value represents the mean ± SD of three independent experiments. * *p* < 0.05, ** *p* < 0.01 *vs.* control.

To probe the apoptotic effects of plumbagin (**1**) and **11a** on cell cycle progression, HT-29 cells were treated once again with these compounds, separately, at different concentrations for 48 h. The cell cycle distribution of the cancer cells was analyzed by flow cytometry, and the sub-G1 phase analyzed by flow cytometry with propidium iodide (PI) staining [28]. The control of the experiment was the untreated cells. As illustrated in Figure 3, only a small fraction of apoptotic cells (0.8%) was detected in the control as well as in plumbagin (**1**) at a low concentration of 2.5 μM. However, as plumbagin (**1**) dosage increased from 0.5 to 2.5 μM, the fraction of apoptotic cell went up substantially from 4.5% to 7.6% in a concentration-dependent manner. As for compound **11a**, the apoptotic effect meagerly laid between 10.7% to 27.8% across the treatment range of 0.5–2.5 μM and induced cell accumulation in the S phase.

### 2.6. Apoptotic Analyses-Annexin V-FITC/PI Double Staining and Flow Cytometry Analyses

**Figure 4:**
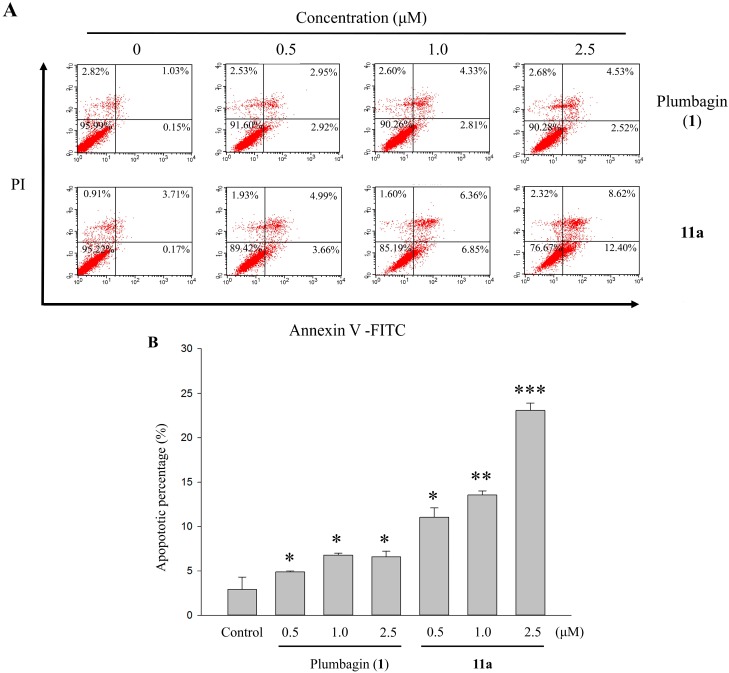
(**A**) The effects of the 48 h treatments with 0–2.5 μM plumbagin (**1**) and **11a** on apoptotic percentage distribution of HT-29 cells by Annexin V-FITC/PI staining. (**B**) The apoptosis rate was calculated by flow cytometry and cell apoptosis was defined in early and late apoptosis treatment with 0–2.5 μM plumbagin (**1**) and **11a** for 48 h. Each value represents the mean ± SD of three independent experiments. * *p* < 0.05, ** *p* < 0.01 and *** *p* < 0.001 *vs.* control.

Quantitative analysis of apoptotic effects of plumbagin (**1**) and **11a** on HT-29 cells was conducted by flow cytometry using Annexin V-FITC and PI double staining. This was to study in depth the bioactivities of plumbagin (**1**) and **11a** against HT-29 cells. Thus, the cancer cells were treated with vehicle alone as control or with one of the two testing compounds at different concentrations (0.5–2.5 μM). After 48 h, the samples were double-stained with Annexin V-FITC and PI [29]. The percentages of cell populations at various stages of apoptosis were exhibited in Figure 4. The total apoptosis rates were 1.08%, 8.65%, 13.21%, and 21.02% at concentrations of 0, 0.5, 1.0, and 2.5 μM of compound **11a**, respectively. Though the data pointed out that the distributions of apoptotic cell death resulting from the treatment of lawsone derivative **11a** were concentration-dependent, this was not the case for plumbagin (**1**). Starting from a dosage of 0.5 μM, compound **11a** induced higher frequency of HT-29 cells apoptosis, as well as cytotoxic effects at both early and late stages. For plumbagin (**1**) though, the only discernible effect was seen at a higher threshold (2.5 μM). We attribute this finding to and confirmed that the superior efficiency of lawsone derivative **11a** in its cytotoxicity and inhibitive function on human colorectal cell proliferation.

## 3. Experimental Section 

### 3.1. General

All chemical reagents of commercial quality were used as received (Sigma-Aldrich, St. Louis, MO, USA) and were used without further purification. Solvents were dried and the synthesized compounds were purified using standard techniques. The progression of reactions was monitored by TLC on aluminum plates coated with silica gel with a fluorescent indicator (Merck, Darmstadt, Germany) unless otherwise stated. Melting points were determined using open capillaries using the Fargo MP-2D apparatus (Prosperous instrument, Chaiyi, Taiwan, ROC) and are reported uncorrected. NMR spectra were recorded using TMS as an internal standard in CDCl_3_ at 500 MHz for ^1^H and at 125 MHz for ^13^C (Bruker Biospin GmbH AVANCE III 500 MHz, Rheinstetten, Germany). The mass spectra were acquired using a Thermo Finnigan model LXQ (Thermo Electron Co., Waltham, MA, USA) ion trap mass spectrometer equipped with ESI source interference and controlled by Xcalibur 2.06 (Thermo Electron Co., Waltham, MA, USA). The mass spectra were acquired in a positive ion mode or a negative ion mode. ESI high-resolution mass spectra (HRMS) were recorded on a Finnigan MAT 95S mass spectrometry (Thermo Fisher Scientific Co. Ltd., Waltham, MA, USA). Column chromatography was performed with silica gel Silia*Flash*^®^ G60 (60–200 μm, Quebec City, QC, Canada) purchased from SiliCycle Inc. (Quebec City, QC, Canada). In general, the reactions were carried out under anhydrous conditions in dry solvent and nitrogen atmosphere. The purity of the compounds was more than 98% based on the analysis of HPLC (Hitachi High-Technologies, Tokyo, Japan) using a RP-C_18_ column (4.6 mm × 250 mm, 5 μm, Merck, Darmstadt, Germany).

### 3.2. General Synthetic Procedure for **5a**,**b**‒**11a**,**b**

A mixture of lawsone (2.87 mmol), NaI (3.16 mmol), triethylamine (3.16 mmol) and alkyl or terpenyl bromide (2.87 mmol) were stirred in DMF (5 mL) at 40 °C for three days. After the reaction was cooled to room temperature, 1 N HCl was added and the solution was poured into a separatory funnel with water, dichloromethane was added and the organic layer was separated from the aqueous layer. The organic layer was collected, dried over anhydrous magnesium sulfate (MgSO_4_) and concentrated under reduced pressure to remove the solvent, and then was subjected to column chromatography (ethyl acetate/*n*-hexane = 1/7) to produce yellow solid compounds, which were purified by recrystallization from *n*-hexane.

*2-Hydroxy-3-methyl-1*,*4-naphthoquinone* (**5a**). The reaction produced **5a** in 45.3% as a yellow solid; mp 173.4–174.1 °C (lit. [30] 173–174 °C). ^1^H-NMR (500 MHz, CDCl_3_) δ_H_ 2.09 (s, 3H, CH_3_), 7.29 (bs, 1H, 2-OH), 7.66 (dt, *J* = 1.2, 7.5 Hz, 1H, H-7), 7.73 (dt, *J* = 1.4, 7.6 Hz, 1H, H-6), 8.06 (dd, *J* = 1.2, 7.7 Hz, 1H, H-8), 8.10 (dd, *J* = 0.7, 7.7 Hz, 1H, H-5); ^13^C-NMR (125 MHz, CDCl_3_) δ_C_ 8.91 (CH_3_), 120.75 (C-3), 126.36 (C-8), 126.97 (C-5), 129.64 (C-9), 133.13 (C-6), 132.94 (C-10), 134.83 (C-7), 153.13 (C-2) 181.19 (C=O), 185.02 (C=O); LC-MS (ESI^−^, *m*/*z*) calculated for C_11_H_8_O_3_: 188.05, found for 186.98 [M − H]^−^; HRMS-ESI: *m*/*z* calculated for C_11_H_8_O_3_: 188.0473 [M]^+^, found for 188.0471.

*2-Methoxy-1*,*4-naphthoquinone* (**5b**). The reaction produced **5b** in 27.0% as a yellow solid; mp 187.0–187.8 °C (lit. [31] 186–189 °C). ^1^H-NMR (500 MHz, CDCl_3_) δ_H_ 3.89 (s, 3H, CH_3_), 6.16 (s, 1H, H-3), 7.70 (dt, *J* = 1.3, 7.5 Hz, 1H, H-7), 7.74 (dt, *J* = 1.3, 7.5 Hz, 1H, H-6), 8.07 (dd, *J* = 1.2, 7.5 Hz, 1H, H-8), 8.11 (dd, *J* = 1.2, 7.5 Hz, 1H, H-5); ^13^C-NMR (125 MHz, CDCl_3_) δ_C_ 56.65 (CH_3_), 110.14 (C-3), 126.43 (C-8), 126.96 (C-5), 131.29 (C-9), 132.28 (C-10), 133.58 (C-6), 134.57 (C-7), 160.67 (C-2), 180.36 (C=O), 185.08 (C=O); LC-MS (ESI^+^, *m*/*z*) calculated for C_11_H_8_O_3_: 188.05, found for 189.09 [M + H]^+^; HRMS-ESI: *m*/*z* calculated for C_11_H_8_O_3_: 188.0473 [M]^+^, found for 188.0476.

*2-Hydroxy-3-butyl-1*,*4-naphthoquinone* (**6a**). The reaction produced **6a** in 36.6% as a yellow solid; mp 97.1–98.0 °C (lit. [32] 93–94 °C). ^1^H-NMR (500 MHz, CDCl_3_) δ_H_ 0.92 (t, *J* = 7.5 Hz, 3H, CH_3_), 1.39 (p, *J* = 7.5 Hz 2H, H-3′), 1.50 (p, *J* = 7.5 Hz, 2H, H-2′), 2.58 (t, *J* = 7.5 Hz, 2H, H-1′), 7.30 (s, 1H, 2-OH), 7.65 (t, *J* = 7.5 Hz, 1H, H-7), 7.73 (t, *J* = 7.5 Hz, 1H, H-6), 8.05 (d, *J* = 7.5 Hz, 1H, H-8), 8.10 (d, *J* = 7.5 Hz, 1H, H-5); ^13^C-NMR (125 MHz, CDCl_3_) δ_C_ 14.12 (CH_3_), 23.09 (C-1′), 29.91 (C-3′), 30.64 (C-2′), 125.04 (C-3), 126.26 (C-8), 126.99 (C-5), 129.67 (C-9), 133.06 (C-6), 133.18 (C-10), 135.04 (C-7), 153.23 (C-2), 181.70 (C=O), 184.96 (C=O); LC-MS (ESI^−^, *m*/*z*) calculated for C_14_H_14_O_3_: 230.09, found for 229.05 [M − H]^−^; HRMS-ESI: *m*/*z* calculated for C_14_H_14_O_3_: 230.0943 [M]^+^, found for 230.0948.

*2-Butyloxy-1*,*4-naphthoquinone* (**6b**). The reaction produced **6b** in 5.5% as a yellow solid; mp 111.5–112.1 °C (lit. [33] 97–98 °C). ^1^H-NMR (500 MHz, CDCl_3_) δ_H_ 0.99 (t, *J* = 7.4 Hz, 3H, CH_3_), 1.53 (p, *J* = 7.5 Hz 2H, H-3′), 1.88 (p, *J* = 7.1 Hz, 2H, H-2′), 4.02 (t, *J* = 6.6 Hz, 2H, H-1′), 6.16 (s, 1H, H-3), 7.70 (dt, *J* = 1.0, 7.5 Hz, 1H, H-7), 7.74 (dt, *J* = 1.0, 7.5 Hz, 1H, H-6), 8.07 (d, *J* = 7.3 Hz, 1H, H-8), 8.11 (d, *J* = 7.3 Hz, 1H, H-5); ^13^C-NMR (125 MHz, CDCl_3_) δ_C_ 13.70 (CH_3_), 19.13 (C-3′), 30.25 (C-2′), 69.39 (C-1′), 110.19 (C-3), 126.11 (C-8), 126.68 (C-5), 131.20 (C-9), 132.04 (C-10), 133.26 (C-6), 134.21 (C-7), 159.93 (C-2), 180.18 (C=O), 185.10 (C=O); LC-MS (ESI^−^, *m*/*z*) calculated for C_14_H_14_O_3_: 230.09, found for 229.52 [M − H]^−^; HRMS-ESI: *m*/*z* calculated for C_14_H_14_O_3_: 230.0943 [M]^+^, found for 230.0941.

*2-Hydroxy-3-octyl-1*,*4-naphthoquinone* (**7a**). The reaction produced **7a** in 36.2% as a yellow solid; mp 91.1–91.6 °C (lit. [34] 88–89 °C). ^1^H-NMR (500 MHz, CDCl_3_) δ_H_ 0.86 (t, *J* = 7.5 Hz, 3H, CH_3_), 1.23–1.36 (m, 8H, H-4′, H-5′, H-6′, H-7′), 1.39 (p, *J* = 8.0 Hz, 2H, H-3′), 1.51 (p, *J* = 7.6 Hz, 2H, H-2′), 2.58 (t, *J* = 7.7 Hz, 2H, H-1′), 7.26 (s, 1H, 2-OH), 7.66 (dt, *J* = 1.0, 7.6 Hz, 1H, H-7), 7.73 (dt, *J* = 1.0, 7.6 Hz, 1H, H-6), 8.05 (d, *J* = 7.5 Hz, 1H, H-8), 8.10 (d, *J* = 7.6 Hz, 1H, H-5); ^13^C-NMR (125 MHz, CDCl_3_) δ_C_ 14.32 (CH_3_), 22.88 (C-1′), 28.53 (C-7′), 29.46 (C-2′), 29.64 (C-3′), 29.92 (C-4′), 30.41 (C-5′), 32.10 (C-6′), 125.09 (C-3), 126.27 (C-8), 127.01 (C-5), 129.69 (C-9), 133.06 (C-6), 133.21 (C-10), 135.04 (C-7), 153.20 (C-2), 181.72 (C=O), 184.94 (C=O); LC-MS (ESI^+^, *m*/*z*) calculated for C_18_H_22_O_3_: 286.16, found for 309.35 [M + Na]^+^; HRMS-ESI: *m*/*z* calculated for C_18_H_22_O_3_: 286.1569 [M]^+^, found for 286.1568.

*2-Octyloxy-1*,*4-naphthoquinone* (**7b**). The reaction produced **7b** in 3.8% as a yellow solid; mp 94.1–95.8 °C. ^1^H-NMR (500 MHz, CDCl_3_) δ_H_ 0.86 (t, *J* = 6.9, 3H, CH_3_), 1.23–1.35 (m, 8H, H-4′, H-5′, H-6′, H-7′), 1.44 (p, *J* = 7.6 Hz, 2H, H-3′), 1.87 (p, *J* = 7.2 Hz, 2H, H-2′), 3.98 (t, *J* = 6.7 Hz, 2H, H-1′), 6.12 (s, 1H, H-3), 7.68 (dt, *J* = 1.5, 7.5 Hz, 1H, H-7), 7.71 (dt, *J* = 1.0, 7.5 Hz, 1H, H-6), 8.05 (dd, *J* = 1.0, 7.0 Hz, 1H, H-8), 8.10 (dd, *J* = 1.5, 7.5 Hz, 1H, H-5); ^13^C-NMR (125 MHz, CDCl_3_) δ_C_ 14.08 (CH_3_), 22.63 (C-7′), 25.85 (C-2′), 28.26 (C-3′), 29.14 (C-4′), 29.20 (C-5′), 31.75 (C-6′), 69.69 (C-1′), 110.19 (C-3), 126.11 (C-8), 126.67 (C-5), 131.20 (C-9), 132.05 (C-10), 133.24 (C-6), 134.20 (C-7), 159.92 (C-2), 180.18 (C=O), 185.09 (C=O); LC-MS (ESI^+^, *m*/*z*) calculated for C_18_H_22_O_3_: 286.16, found for 309.25 [M + Na]^+^; HRMS-ESI: *m*/*z* calculated for C_18_H_22_O_3_: 286.1569 [M]^+^, found for 286.1564.

*2-Hydroxy-3-dodectyl-1*,*4-naphthoquinone* (**8a**). The reaction produced **8a** in 56.3% as a yellow solid; mp 88.4–88.9 °C (lit. [35] 85–87 °C). ^1^H-NMR (500 MHz, CDCl_3_) δ_H_ 0.86 (t, *J* = 7.0 Hz, 3H, CH_3_), 1.23–1.35 (m, 18H, H-3′, H-4′, H-5′, H-6′, H-7′, H-8′, H-9′, H-10′, H-11′), 1.51 (p, *J* = 7.5 Hz, 2H, H-2′), 2.58 (t, *J* = 7.7 Hz, 2H, H-1′), 7.26 (s, 1H, 2-OH), 7.61 (dt, *J* = 0.8, 7.5 Hz, 1H, H-7), 7.73 (dt, *J* = 0.8, 7.5 Hz, 1H, H-6), 8.00 (d, *J* = 7.5 Hz, 1H, H-8), 8.05 (d, *J* = 7.5 Hz, 1H, H-5); ^13^C-NMR (125 MHz, CDCl_3_) δ_C_ 14.33 (CH_3_), 22.91 (C-1′), 23.62 (C-11′), 28.53 (C-2′), 29.57 (C-3′), 29.68 (C-4′), 29.80 (C-5′), 29.86 (C-6′), 29.88 (C-7′), 29.92 (C-8′), 30.02 (C-9′), 32.14 (C-10′), 125.09 (C-3), 126.26 (C-8), 127.00 (C-5), 129.69 (C-9), 133.05 (C-6), 133.21 (C-10), 135.04 (C-7), 153.20 (C-2), 181.71 (C=O), 184.93 (C=O); LC-MS (ESI^−^, *m*/*z*) calculated for C_22_H_30_O_3_: 342.22, found for 341.38 [M − H]^−^; HRMS-ESI: *m*/*z* calculated for C_22_H_30_O_3_: 342.2195 [M]^+^, found for 342.2189.

*2-Dodectyloxy-1*,*4-naphthoquinone* (**8b**). The reaction produced **8b** in 6.9% as a yellow solid; mp 96.1–96.7 °C. ^1^H-NMR (500 MHz, CDCl_3_) δ_H_ 0.86 (t, *J* = 7.0 Hz, 3H, CH_3_), 1.24–1.33 (m, 16H, H-4′, H-5′, H-6′, H-7′, H-8′, H-9′, H-10′, H-11′), 1.44 (p, *J* = 7.5 Hz, 2H, H-3′), 1.87 (p, *J* = 7.1 Hz, 2H, H-2′), 3.98 (t, *J* = 6.7 Hz, 2H, H-1′), 6.13 (s, 1H, H-3), 7.68 (t, *J* = 7.3 Hz, 1H, H-7), 7.72 (t, *J* = 7.3 Hz, 1H, H-6), 8.05 (d, *J* = 7.4 Hz, 1H, H-8), 8.10 (d, *J* = 7.5 Hz, 1H, H-5); ^13^C-NMR (125 MHz, CDCl_3_) δ_C_ 14.33 (CH_3_), 22.90 (C-7′), 26.06 (C-2′), 28.48 (C-3′), 29.46 (C-4′), 29.56 (C-5′), 29.69 (C-6′), 29.77 (C-7′), 29.83 (C-8′), 29.84 (C-9′), 32.13 (C-10′), 69.90 (C-1′), 110.40 (C-3), 126.32 (C-8), 126.89 (C-5), 131.42 (C-9), 132.26 (C-10), 133.46 (C-6), 134.42 (C-7), 160.13 (C-2), 180.40 (C=O), 185.31 (C=O); LC-MS (ESI^−^, *m*/*z*) calculated for C_22_H_30_O_3_: 342.22, found for 341.30 [M − H]^−^; HRMS-ESI: *m*/*z* calculated for C_22_H_30_O_3_: 342.2195 [M]^+^, found for 342.2197.

*2-Hydroxy-3-isoprenyl-1*,*4-naphthoquinone* (**9a**). The reaction produced **9a** in 58.2% as a yellow solid; mp 142.5–144.0 °C (lit. [36] 141–143 °C). ^1^H-NMR (500 MHz, CDCl_3_) δ_H_ 1.66 (s, 3H, CH_3_), 1.77 (s, 3H, CH_3_), 3.29 (d, *J* = 7.4 Hz, 2H, H-1′), 5.17–5.21 (m, 2H, H-2′), 7.27 (s, 1H, 2-OH), 7.66 (dt, *J* = 1.1, 7.4 Hz, 1H, H-7), 7.73 (dt, *J* = 1.4, 7.6 Hz, 1H, H-6), 8.05 (dd, *J* = 1.0, 7.4 Hz, 1H, H-8), 8.10 (dd, *J* = 1.0, 7.7 Hz, 1H, H-5); ^13^C-NMR (125 MHz, CDCl_3_) δ_C_ 18.12 (CH_3_), 22.84 (CH_3_), 25.98 (C-1′), 119.85 (C-3), 123.68 (C-2′), 126.28 (C-8), 127.01 (C-5), 129.65 (C-9), 133.09 (C-6), 133.14 (C-10), 134.09 (C-3ʹ), 135.08 (C-7), 152.87 (C-2), 181.93 (C=O), 184.79 (C=O); LC-MS (ESI^−^, *m*/*z*) calculated for C_15_H_14_O_3_: 242.09, found for 241.16 [M − H]^–^; HRMS-ESI: *m*/*z* calculated for C_15_H_14_O_3_: 242.0943 [M]^+^, found for 242.0948.

*2-Isoprenyloxy-1*,*4-naphthoquinone* (**9b**). The reaction produced **9b** in 4.8% as a yellow solid; mp 158.0–159.6 °C (lit. [37] 150–151 °C). ^1^H-NMR (500 MHz, CDCl_3_) δ_H_ 1.74 (s, 3H, CH_3_), 1.79 (s, 3H, CH_3_), 4.57 (d, *J* = 7.8 Hz, 2H, H-1′), 5.46–5.49 (m, 2H, H-2′), 6.14 (s, 1H, H-3), 7.68 (dt, *J* = 1.5, 7.5 Hz, 1H, H-7), 7.72 (dt, *J* = 1.5, 7.5 Hz, 1H, H-6), 8.06 (dd, *J* = 1.5, 7.5 Hz, 1H, H-8), 8.10 (dd, *J* = 1.5, 7.5 Hz, 1H, H-5); ^13^C-NMR (125 MHz, CDCl_3_) δ_C_ 18.58 (CH_3_), 26.05 (CH_3_), 66.63 (C-1′), 110.73 (C-3), 117.41 (C-2′), 126.33 (C-8), 126.92 (C-5), 131.41 (C-9), 132.26 (C-10), 133.46 (C-6), 134.43 (C-7), 140.73 (C-3′), 159.80 (C-2), 180.51 (C=O), 185.28 (C=O); LC-MS (ESI^−^, *m*/*z*) calculated for C_15_H_14_O_3_: 242.09, found for 241.23 [M − H]^−^; HRMS-ESI: *m*/*z* calculated for C_15_H_14_O_3_: 242.0943 [M]^+^, found for 242.0941.

*2-Hydroxy-3-geranyl-1*,*4-naphthoquinone* (**10a**). The reaction produced **10a** in 50.8% as a yellow solid; mp 112.0–112.5 °C [38]. ^1^H-NMR (500 MHz, CDCl_3_) δ_H_ 1.59 (s, 3H, CH_3_), 1.65 (s, 3H, CH_3_), 1.81 (s, 3H, CH_3_), 1.98–2.01 (m, 2H, H-5′), 2.06–2.09 (m, 2H, H-6′), 3.34 (d, *J* = 7.5 Hz, 2H, H-1′), 5.06–5.09 (m, 1H, H-7′), 5.23 (dt, *J* = 1.0, 7.5 Hz, 1H, H-2′), 7.31 (s, 1H, 2-OH), 7.70 (dt, *J* = 1.0, 7.5 Hz, 1H, H-7), 7.78 (dt, *J* = 1.0, 7.5 Hz, 1H, H-6), 8.10 (dd, *J* = 1.5, 7.5 Hz, 1H, H-8), 8.15 (dd, *J* = 1.0, 7.5 Hz, 1H, H-5); ^13^C-NMR (125 MHz, CDCl_3_) δ_C_ 16.47 (CH_3_), 17.88 (CH_3_), 22.77 (CH_3_), 25.87 (C-1′), 26.82 (C-6′), 39.96 (C-5′), 119.67 (C-3), 123.76 (C-2′), 124.41 (C-7′), 126.28 (C-8), 127.00 (C-5), 129.65 (C-8′), 131.61 (C-9), 133.08 (C-6), 133.15 (C-10), 135.08 (C-7), 137.61 (C-3′), 152.88 (C-2), 181.94 (C=O), 184.74 (C=O); LC-MS (ESI^−^, *m*/*z*) calculated for C_20_H_22_O_3_: 310.16, found for 309.12 [M − H]^−^; HRMS-ESI: *m*/*z* calculated for C_20_H_22_O_3_: 310.1569 [M]^+^, found for 310.1572.

*2-Geranyloxy-1*,*4-naphthoquinone* (**10b**). The reaction produced **10b** in 5.2% as a pale yellow liquid. ^1^H-NMR (500 MHz, CDCl_3_) δ_H_ 1.59 (s, 3H, CH_3_), 1.65 (s, 3H, CH_3_), 1.81 (s, 3H, CH_3_), 1.99–2.02 (m, 2H, H-5′), 2.07–2.10 (m, 2H, H-6′), 4.58 (d, *J* = 6.6 Hz, 2H, H-1´), 5.06–5.10 (m, 1H, H-7´), 5.46 (dt, *J* = 0.8, 6.6 Hz, 1H, H-2′), 6.14 (s, 1H, H-3), 7.68 (dt, *J* = 1.0, 7.5 Hz, 1H, H-7), 7.74 (dt, *J* = 1.0, 7.5 Hz, 1H, H-6), 8.06 (dd, *J* = 1.5, 7.5 Hz, 1H, H-8), 8.10 (dt, *J* = 1.0, 7.5 Hz, 1H, H-5); ^13^C-NMR (125 MHz, CDCl_3_) δ_C_ 16.47 (CH_3_), 17.68 (CH_3_), 25.70 (CH_3_), 26.71 (C-6′), 39.65 (C-5′), 66.51 (C-1′), 110.56 (C-3), 117.22 (C-2′), 124.37 (C-7′), 126.23 (C-8), 126.88 (C-5), 131.18 (C-8′), 132.06 (C-9), 133.18 (C-6), 134.26 (C-10), 135.73 (C-7), 141.56 (C-3′), 159.56 (C-2), 180.29 (C=O), 185.06 (C=O); LC-MS (ESI^−^, *m*/*z*) calculated for C_20_H_22_O_3_: 310.16, found for 309.06 [M − H]^−^; HRMS-ESI: *m*/*z* calculated for C_20_H_22_O_3_: 310.1569 [M]^+^, found for 310.1574.

*2-Hydroxy-3-farnesyl-1*,*4-naphthoquinone* (**11a**). The reaction produced **11a** in 35.2% as a yellow solid; mp 84.4–84.9 °C (lit. [39] 68–69 °C). ^1^H-NMR (500 MHz, CDCl_3_) δ_H_ 1.54 (s, 3H, CH_3_), 1.54 (s, 6H, CH_3_), 1.77 (s, 3H, CH_3_), 1.92–1.95 (m, 2H, H-5′), 1.97–2.00 (m, 4H, H-9′, H-10′) 2.02–2.07 (m, 2H, H-6′), 3.30 (d, *J* = 7.5 Hz, 2H, H-1′), 5.00–5.05 (m, 2H, H-7′, H-12′) 5.20 (t, 1H, *J* = 7.3 Hz, 1H, H-2′), 7.28 (s, 1H, 2-OH), 7.65 (t, *J* = 7.5 Hz, 1H, H-7), 7.73 (t, *J* = 7.5 Hz, 1H, H-6), 8.05 (d, *J* = 7.5 Hz, 1H, H-8), 8.11 (d, *J* = 7.5 Hz, 1H, H-5); ^13^C-NMR (125 MHz, CDCl_3_) δ_C_ 16.22 (CH_3_), 16.48 (CH_3_), 17.86 (CH_3_), 22.77 (C-1′), 25.90 (CH_3_), 26.69 (C-6′), 26.94 (C-11′), 39.89 (C-5′), 39.95 (C-9′), 119.69 (C-3), 123.75 (C-2′), 124.24 (C-7′), 124.57 (C-11′), 126.27 (C-8), 127.00 (C-5), 129.66 (C-9), 131.43 (C-10), 133.06 (C-6), 133.14 (C-12′), 135.06 (C-7), 135.24 (C-8′), 137.60 (C-3′), 152.89 (C-2), 181.92 (C=O), 184.74 (C=O); LC-MS (ESI^−^, *m*/*z*) calculated for C_25_H_30_O_3_: 378.22, found for 377.23 [M − H]^−^; HRMS-ESI: *m*/*z* calculated for C_25_H_30_O_3_: 378.2195 [M]^+^, found for 378.2193.

*2-Farnesyloxy-1*,*4-naphthoquinone* (**11b**). The reaction produced **11b** in 7.4% as a yellow liquid. ^1^H-NMR (500 MHz, CDCl_3_) δ_H_ 1.56 (s, 3H, CH_3_), 1.58 (s, 6H, CH_3_), 1.74 (s, 3H, CH_3_), 1.92–1.95 (m, 2H, H-5′), 2.00–2.03 (m, 2H, H-6′) 2.08–2.12 (m, 4H, H-9′, H-10′), 4.60 (d, *J* = 6.6 Hz, 2H, H-1′), 5.04–5.08 (m, 2H, H-7′, H-12′) 5.47 (dt, 1H, *J* = 0.8, 6.6 Hz, 1H, H-2′), 6.14 (s, 1H, H-3), 7.69 (dt, *J* = 1.5, 7.5 Hz, 1H, H-7), 7.72 (dt, *J* = 1.5, 7.5 Hz, 1H, H-6), 8.06 (dd, *J* = 1.5, 7.5 Hz, 1H, H-8), 8.10 (dd, *J* = 1.5, 7.5 Hz, 1H, H-5); ^13^C-NMR (125 MHz, CDCl_3_) δ_C_ 16.05 (CH_3_), 16.88 (CH_3_), 17.68 (CH_3_), 25.70 (CH_3_), 26.09 (C-6′), 26.71 (C-11′), 39.53 (C-5′), 39.67 (C-9′), 66.50 (C-1′), 110.55 (C-3), 116.91 (C-2′), 123.39 (C-7′), 124.28 (C-11′), 126.12 (C-8), 126.70 (C-5), 131.20 (C-9), 131.33 (C-10), 132.04 (C-12′), 133.24 (C-6), 134.21 (C-7), 135.72 (C-8′), 143.63 (C-3′), 159.58 (C-2), 180.31 (C=O), 185.04 (C=O); LC-MS (ESI^−^, *m*/*z*) calculated for C_25_H_30_O_3_: 378.22, found for 377.26 [M − H]^−^; HRMS-ESI: *m*/*z* calculated for C_25_H_30_O_3_: 378.2195 [M]^+^, found for 378.2190.

### 3.3. 5-Hydroxy-1,4-Naphthoquinone (Juglone, **3**)

A suspension of 1,5-dihydroxynaphthene (1 g, 6.25 mmol) in THF/H_2_O (50 mL, 1/1, *v*/*v*) was placed in a 100 mL, three-necked flask fitted with a mechanical stirrer and a gas inlet tube and a strong current of air was bubbled through it. Then, periodic acid (3.12 g, 13.7 mmol) was added with vigorous stirring at room temperature in the dark over 1 h, and the resultant mixture was stirred for 4 h. The mixture was filtered, washed with acetonitrile, and the solvent removed under reduced pressure. The crude product was purified using silica gel with ethyl acetate/*n*-hexane (1:5) as the solvent to afford 5-hydroxy-1,4-naphthoquinone (juglone, **3**) (735 mg, 4.22 mmol, 67.6%) as a brown-red solid; mp 146.0–148.2 °C (lit. [40] 152 °C). ^1^H-NMR (500 MHz, CDCl_3_) δ_H_ 6.93 (s, 2H, H-2, H-3), 7.26 (dd, *J* = 1.5, 7.5 Hz, 1H, H-6), 7.59–7.63 (m, 2H, H-7, H-8), 11.88 (s, 1H, 5-OH); ^13^C-NMR (125 MHz, CDCl_3_) δ_C_ 114.97 (C-10), 119.15 (C-6), 124.49 (C-8), 131.76 (C-9), 136.56 (C-7), 138.65 (C-3), 139.59 (C-2), 161.45 (C-5), 184.25 (C=O), 190.28 (C=O); LC-MS (ESI^−^, *m*/*z*) calculated for C_10_H_6_O_3_: 174.03, found for 173.10 [M − H]^−^; HRMS-ESI: *m*/*z* calculated for C_10_H_6_O_3_: 174.0317 [M]^+^, found for 174.0313.

### 3.4. General Proceed Amination of Juglone with Alkyl Amine

A suspension of juglone (200 mg, 1.15 mmol) and alkyl amine (1.15 mmole) in 50 mL of ethanol was stirred for 1 h under reflux. The mixture was poured into water, 2 mL of 1 N hydrochloric acid was added, the precipitate was filtered off, washed with water, dried, and dissolved in chloroform, and the solution was applied to a column charged with silica gel. The column was eluted with ethyl acetate/*n*-hexane (1/5) to give the 2-amination and 3-amination products, respectively.

*2-Butylamino-5-hydroxy-1*,*4-naphthoquinone* (**13a**). The reaction produced **13a** in 13.9% as an orange red solid; mp 154.6–155.4 °C (lit. [41] 156–157 °C). ^1^H-NMR (500 MHz, CDCl_3_) δ_H_ 0.96 (t, *J* = 7.4 Hz, 3H, CH_3_), 1.43 (p, *J* = 7.5 Hz, 2H, H-2′), 1.67 (p, *J* = 7.5 Hz, 2H, H-3′), 3.17 (q, *J* = 6.9 Hz, 2H, H-1′), 5.61 (s, 1H, H-3), 6.04 (s, 1H, -NH), 7.22 (dd, *J* = 0.8, 8.3 Hz, 1H, H-8), 7.44 (t, *J* = 8.0 Hz, 1H, H-7), 7.56 (dd, 1H, *J* = 0.8, 7.6 Hz, H-6), 13.08 (s, 1H, 5-OH); ^13^C-NMR (125 MHz, CDCl_3_) δ_C_ 13.90 (CH_3_), 20.40 (C-2′), 30.43 (C-3′), 42.63 (C-1′), 99.81 (C-3), 115.19 (C-9), 119.25 (C-6), 126.18 (C-8), 130.70 (C-10), 134.05 (C-7), 148.90 (C-2), 161.33 (C-5), 181.41 (C=O), 189.14 (C=O); LC-MS (ESI^+^, *m*/*z*) calculated for C_14_H_15_NO_3_: 245.11, found for 268.24 [M + Na]^+^; HRMS-ESI: *m*/*z* calculated for C_14_H_16_O_3_N: 246.1125 [M + H]^+^, found for 246.1120.

*3-Butylamino-5-hydroxy-1*,*4-naphthoquinone* (**13b**). The reaction produced **13b** in 6.9% as an orange red solid; mp 125.3–126.5 °C (lit. [41] 111–112 °C). ^1^H-NMR (500 MHz, CDCl_3_) δ_H_ 0.96 (t, *J* = 7.5 Hz, 3H, CH_3_), 1.42 (h, *J* = 7.5 Hz, 2H, H-3′), 1.67 (p, *J* = 7.5 Hz, 2H, H-2′), 3.17 (q, *J* = 6.9 Hz, 2H, H-1′), 5.70 (s, 1H, H-3), 5.85 (bs, 1H, -NH), 7.12 (dd, *J* = 2.3, 7.0 Hz, 1H, H-8), 7.57–7.62 (m, 2H, H-6, H-7), 11.52 (s, 1H, 5-OH); ^13^C-NMR (125 MHz, CDCl_3_) δ_C_ 13.91 (CH_3_), 20.42 (C-2′), 30.45 (C-3′), 42.56 (C-1′), 101.50 (C-3), 114.31 (C-9), 118.95 (C-6), 122.37 (C-8), 133.82 (C-10), 137.91 (C-7), 147.97 (C-2), 161.89 (C-5), 182.42 (C=O), 186.36 (C=O); LC-MS (ESI^+^, *m*/*z*) calculated for C_14_H_15_NO_3_: 245.11, found for 268.29 [M + Na]^+^; HRMS-ESI: *m*/*z* calculated for C_14_H_16_O_3_N: 246.1125 [M + H]^+^, found for 246.1121.

*2-Hexylamino-5-hydroxy-1*,*4-naphthoquinone* (**14a**). The reaction produced **14a** in 15.1% as an orange red solid; mp 126.0–127.5 °C. ^1^H-NMR (500 MHz, CDCl_3_) δ_H_ 0.88 (t, *J* = 7.5 Hz, 3H, CH_3_), 1.30–1.32 (m, 4H, H-4′, H-5′), 1.36–1.40 (m, 2H, H-3′), 1.67 (p, *J* = 7.3 Hz, 2H, H-2′), 3.16 (q, *J* = 6.8 Hz, 2H, H-1′), 5.60 (s, 1H, H-3), 6.05 (bs, 1H, -NH), 7.22 (d, *J* = 8.4 Hz, 1H, H-8), 7.44 (t, *J* = 7.9 Hz, 1H, H-7), 7.56 (d, *J* = 7.2 Hz, 1H, H-6), 13.08 (s, 1H, 5-OH); ^13^C-NMR (125 MHz, CDCl_3_) δ_C_ 14.19 (CH_3_), 22.72 (C-5′), 26.88 (C-2′), 28.38 (C-3′), 31.59 (C-4′), 42.93 (C-1′), 99.78 (C-3), 115.18 (C-9), 119.24 (C-6), 126.16 (C-8), 130.68 (C-10), 134.03 (C-7), 148.87 (C-2), 161.31 (C-5), 181.40 (C=O), 189.12 (C=O); LC-MS (ESI^+^, *m*/*z*) calculated for C_16_H_19_NO_3_: 273.14, found for 296.29 [M + Na]^+^; HRMS-ESI: *m*/*z* calculated for C_16_H_20_O_3_N: 274.1438 [M+H]^+^, found for 274.1435

*3-Hexylaminp-5-hydroxy-1*,*4-naphthoquinone* (**14b**). The reaction produced **14b** in 24.1% as an orange red solid; mp 110.2–111.1 °C. ^1^H-NMR (500 MHz, CDCl_3_) δ_H_ 0.88 (t, *J* = 6.4 Hz, 3H, CH_3_), 1.31–1.32 (m, 4H, H-4′, H-5′), 1.39 (p, *J* = 6.9 Hz, 2H, H-3′), 1.69 (p, *J* = 7.3 Hz, 2H, H-2′), 3.16 (q, *J* = 6.6 Hz, 2H, H-1′), 5.69 (s, 1H, H-2), 5.87 (bs, 1H, -NH), 7.11 (dd, *J* = 2.0, 7.2 Hz, 1H, H-6), 7.57–7.61 (m, 2H, H-7, H-8), 11.52 (s, 1H, 5-OH); ^13^C-NMR (125 MHz, CDCl_3_) δ_C_ 13.97 (CH_3_), 22.50 (C-5′), 26.68 (C-2′), 28.17 (C-3′), 31.38 (C-4′), 42.63 (C-1′), 101.24 (C-2), 114.07 (C-9), 118.71 (C-6), 122.14 (C-8), 133.58 (C-10), 137.67 (C-7), 147.73 (C-3), 161.66 (C-5), 182.18 (C=O), 186.11 (C=O); LC-MS (ESI^+^, *m*/*z*) calculated for C_16_H_19_NO_3_: 273.14, found for 296.26 [M + Na]^+^; HRMS-ESI: *m*/*z* calculated for C_16_H_20_O_3_N: 274.1438 [M + H]^+^, found for 274.1433.

*2-Octylamino-5-hydroxy-1*,*4-naphthoquinone* (**15a**). The reaction produced **15a** in 14.1% as an orange red solid; mp 128.2–129.5 °C. ^1^H-NMR (500 MHz, CDCl_3_) δ_H_ 0.87 (t, *J* = 6.6 Hz, 3H, CH_3_), 1.26–1.29 (m, 6H, H-5′, H-6′, H-7′), 1.36–1.39 (m, 4H, H-3′, H-4′), 1.67 (p, *J* = 7.3 Hz, 2H, H-2′), 3.16 (q, *J* = 6.6 Hz, 2H, H-1′), 5.60 (s, 1H, H-3), 6.04 (bs, 1H, -NH), 7.22 (d, *J* = 8.0 Hz, 1H, H-8), 7.44 (t, *J* = 7.9 Hz, 1H, H-7), 7.57 (d, *J* = 7.5 Hz, 1H, H-6), 13.08 (s, 1H, 5-OH); ^13^C-NMR (125 MHz, CDCl_3_) δ_C_ 14.29 (CH_3_), 22.83 (C-7′), 27.22 (C-2′), 28.43 (C-3′), 29.35 (C-4′), 29.92 (C-5′), 31.95 (C-6′), 42.94 (C-1′), 99.80 (C-3), 115.20 (C-9), 119.25 (C-6), 126.18 (C-8), 130.70 (C-10), 134.04 (C-7), 148.88 (C-2), 161.33 (C-5), 181.42 (C=O), 189.14 (C=O); LC-MS (ESI^+^, *m*/*z*) calculated for C_18_H_23_NO_3_: 301.17, found for 324.34 [M + Na]^+^; HRMS-ESI: *m*/*z* calculated for C_18_H_24_O_3_N: 302.1751 [M + H]^+^, found for 302.1746.

*3-Octylamino-5-hydroxy-1*,*4-naphthoquinone* (**15b**). The reaction produced **15b** in 31.4% as an orange red solid; mp 119.5–120.3 °C. ^1^H-NMR (500 MHz, CDCl_3_) δ_H_ 0.87 (t, *J* = 6.3 Hz, 3H, CH_3_), 1.23–1.29 (m, 6H, H-5′, H-6′, H-7′), 1.37–1.39 (m, 2H, H-4′), 1.65–1.70 (m, 4H, H-2′, H-3′), 3.16 (q, *J* = 6.4 Hz, 2H, H-1′), 5.70 (s, 1H, H-2), 5.87 (bs, 1H, -NH), 7.11 (d, *J* = 7.3, 1H, H-8), 7.57–7.61 (m, 2H, H-6, H-7), 11.52 (s, 1H, 5-OH); ^13^C-NMR (125 MHz, CDCl_3_) δ_C_ 14.29 (CH_3_), 22.84 (C-7′), 27.24 (C-2′), 28.45 (C-3′), 29.36 (C-4′), 29.42 (C-5′), 31.96 (C-6′), 42.90 (C-1′), 101.46 (C-2), 114.31 (C-9), 118.98 (C-6), 122.40 (C-8), 133.81 (C-10), 137.93 (C-7), 148.02 (C-3), 161.93 (C-5), 182.40 (C=O), 186.33 (C=O); LC-MS (ESI^+^, *m*/*z*) calculated for C_18_H_23_NO_3_: 301.17, found for 324.36 [M + Na]^+^; HRMS-ESI: *m*/*z* calculated for C_18_H_24_O_3_N: 302.1751 [M + H]^+^, found for 302.1749.

*2-Decylamino-5-hydroxy-1*,*4-naphthoquinone* (**16a**). The reaction produced **16a** in 19.2% as an orange red solid; mp 124.5–125.7 °C. ^1^H-NMR (500 MHz, CDCl_3_) δ_H_ 0.86 (t, *J* = 6.8 Hz, 3H, CH_3_), 1.23–1.30 (m, 8H, H-6′, H-7′, H-8′, H-9′), 1.35–1.40 (m, 6H, H-3′, H-4′, H-5′), 1.67 (p, *J* = 7.3 Hz, 2H, H-2′), 3.17 (q, *J* = 6.7 Hz, 2H, H-1′), 5.61 (s, 1H, H-3), 6.05 (bs, 1H, -NH), 7.22 (dd, *J* = 2.2, 7.3 Hz, 1H, H-8), 7.44 (dt, 1H, *J* = 1.0, 7.5 Hz, H-7), 7.56 (dd, 1H, *J* = 1.0, 7.5 Hz, H-6), 13.08 (s, 1H, 5-OH); ^13^C-NMR (125 MHz, CDCl_3_) δ_C_ 14.09 (CH_3_), 22.65 (C-9′), 26.98 (C-2′), 28.19 (C-3′), 29.20 (C-4′), 29.25 (C-5′), 29.46 (C-6′), 29.47 (C-7′), 31.85 (C-8′), 42.71 (C-1′), 99.57 (C-3), 114.97 (C-9), 119.02 (C-6), 125.95 (C-8), 130.47 (C-10), 133.82 (C-7), 148.66 (C-2), 161.10 (C-5), 181.20 (C=O), 188.91 (C=O); LC-MS (ESI^+^, *m*/*z*) calculated for C_20_H_29_NO_3_: 329.20, found for 352.47 [M + Na]^+^; HRMS-ESI: *m*/*z* calculated for C_20_H_28_O_3_N: 330.2064 [M + H]^+^, found for 330.2058.

*3-Decylamino-5-hydroxy-1*,*4-naphthoquinone* (**16b**). The reaction produced **16b** in 39.5% as an orange red solid; mp 95.8–96.9 °C. ^1^H-NMR (500 MHz, CDCl_3_) δ_H_ 0.86 (t, *J* = 6.8 Hz, 3H, CH_3_), 1.23–1.31 (m, 10H, H-5′, H-6′, H-7′, H-8′, H-9′), 1.35–1.40 (m, 4H, H-3′, H-4′,), 1.67 (p, *J* = 7.3 Hz, 2H, H-2′′), 3.16 (q, *J* = 6.7 Hz, 2H, H-1′), 5.68 (s, 1H, H-2), 5.86 (bs, 1H, -NH), 7.11 (dd, *J* = 2.2, 7.3 Hz, 1H, H-8), 7.57–7.61 (m, 2H, H-6, H-7), 11.51 (s, 1H, 5-OH); ^13^C-NMR (125 MHz, CDCl_3_) δ_C_ 14.32 (CH_3_), 22.88 (C-9′), 27.24 (C-2′), 28.44 (C-3′), 29.45 (C-4′), 29.48 (C-5′), 29.70 (C-6′), 29.92 (C-7′), 32.08 (C-8′), 42.89 (C-1′), 101.45 (C-2), 114.30 (C-9), 118.97 (C-6), 122.39 (C-8), 133.80 (C-10), 137.91 (C-7), 148.02 (C-3), 161.92 (C-5), 182.38 (C=O), 186.31 (C=O); LC-MS (ESI^+^, *m*/*z*) calculated for C_20_H_29_NO_3_: 329.20, found for 330.26 [M + H]^+^; HRMS-ESI: *m*/*z* calculated for C_20_H_28_O_3_N: 330.2064 [M + H]^+^, found for 330.2059.

*2-Dodecylamino-5-hydroxy-1*,*4-naphthoquinone* (**17a**). The reaction produced **17a** in 15.5% as an orange red solid; mp 121.8–122.2 °C. ^1^H-NMR (500 MHz, CDCl_3_) δ_H_ 0.86 (t, *J* = 6.8 Hz, 3H, CH_3_), 1.24–1.31 (m, 14H, H-5′, H-6′, H-7′, H-8′, H-9′, H-10′, H-11′), 1.34–1.40 (m, 4H, H-3′, H-4′), 1.67 (p, *J* = 7.3 Hz, 2H, H-2′), 3.16 (q, *J* = 6.6 Hz, 2H, H-1′), 5.60 (s, 1H, H-3), 6.05 (bs, 1H, -NH), 7.22 (d, *J* = 8.4 Hz, 1H, H-8), 7.44 (t, 1H, *J* = 7.9 Hz, H-7), 7.56 (d, 1H, *J* = 7.5 Hz, H-6), 13.08 (s, 1H, 5-OH); ^13^C-NMR (125 MHz, CDCl_3_) δ_C_ 14.34 (CH_3_), 22.90 (C-11′), 27.22 (C-2′), 28.42 (C-3′), 29.44 (C-4′), 29.55 (C-5′), 29.69 (C-6′), 29.75 (C-7′), 29.83 (C-8′), 29.92 (C-9′), 32.12 (C-10′), 42.94 (C-1′), 99.80 (C-3), 115.20 (C-9), 119.25 (C-6), 126.18 (C-8), 130.70 (C-10), 134.04 (C-7), 148.88 (C-2), 161.33 (C-5), 181.42 (C=O), 189.13 (C=O); LC-MS (ESI^+^, *m*/*z*) calculated for C_22_H_31_NO_3_: 357.23, found for 380.44 [M + Na]^+^; HRMS-ESI: *m*/*z* calculated for C_22_H_32_O_3_N: 358.2377 [M + H]^+^, found for 358.2371.

*3-Dodecylamino-5-hydroxy-1*,*4-naphthoquinone* (**17b**). The reaction produced **17b** in 24.6% as an orange red solid; mp 98.0–98.5 °C. ^1^H-NMR (500 MHz, CDCl_3_) δ_H_ 0.86 (t, *J* = 6.7 Hz, 3H, CH_3_), 1.24–1.31 (m, 12H, H-6′, H-7′, H-8′, H-9′, H-10′, H-11′), 1.35–1.39 (m, 4H, H-4′, H-5′), 1.65–1.70 (m, 4H, H-2′, H-3′), 3.15 (t, *J* = 6.1 Hz, 2H, H-1′), 5.70 (s, 1H, H-2), 5.87 (bs, 1H, -NH), 7.11 (d, *J* = 7.4 Hz, 1H, H-8), 7.57–7.61 (m, 2H, H-6, H-7), 11.52 (s, 1H, 5-OH); ^13^C-NMR (125 MHz, CDCl_3_) δ_C_ 14.34 (CH_3_), 22.90 (C-11′), 27.24 (C-2′), 28.44 (C-3′), 29.45 (C-4′), 29.55 (C-5), 29.70 (C-6′), 29.76 (C-7′), 29.83 (C-8′), 29.92 (C-9′), 32.13 (C-10′), 42.87 (C-1′), 101.49 (C-2), 114.32 (C-9), 118.95 (C-6), 122.37 (C-8), 133.83 (C-10), 137.91 (C-7), 147.97 (C-3), 161.90 (C-5), 182.42 (C=O), 186.34 (C=O); LC-MS (ESI^+^, *m*/*z*) calculated for C_22_H_31_NO_3_: 357.23, found for 380.46 [M + Na]^+^; HRMS-ESI: *m*/*z* calculated for C_22_H_32_O_3_N: 358.2377 [M + H]^+^, found for 358.2374.

*2-[(2-Aminoethyl)-morpholine]-5-hydroxy-1*,*4-naphthoquinone* (**18a**). The reaction produced **18a** in 38.9% as a red solid; mp 122.0–122.5 °C. ^1^H-NMR (500 MHz, CDCl_3_) δ_H_ 2.48 (t, *J* = 4.2 Hz, 4H, H-2′′, H-6′′), 2.67 (t, *J* = 6.0 Hz, 2H, H-2′), 3.22 (q, *J* = 5.7 Hz, 2H, H-1′), 3.72 (t, *J* = 4.6 Hz, 4H, H-3′′, H-5′′), 5.59 (s, 1H, H-3), 6.66 (bs, 1H, -NH), 7.22 (dd, *J* = 0.9, 8.4 Hz, 1H, H-8), 7.45 (t, *J* = 7.9 Hz, 1H, H-7), 7.57 (dd, *J* = 0.9, 7.2 Hz, 2H, H-6), 13.05 (s, 1H, 5-OH); ^13^C-NMR (125 MHz, CDCl_3_) δ_C_ 38.68 (C-1′), 53.39 (C-2′′, C-6′′), 55.68 (C-2′), 67.10 (C-3′′, C-5′′), 100.12 (C-2), 115.18 (C-9), 119.22 (C-6), 126.13 (C-8), 130.76 (C-10), 134.13 (C-7), 148.89 (C-3), 161.32 (C-5), 181.28 (C=O), 189.17 (C=O); LC-MS (ESI^−^, *m*/*z*) calculated for C_16_H_18_N_2_O_4_: 302.13, found for 301.08 [M − H]^−^; HRMS-ESI: *m*/*z* calculated for C_16_H_18_N_2_O_4_: 302.1267 [M]^+^, found for 302.1264.

*3-[(2-Aminoethyl)-morpholine]-5-hydroxy-1*,*4-naphthoquinone* (**18b**). The reaction produced **18b** in 16.8% as a red solid; mp 150.2–151.4 °C. ^1^H-NMR (500 MHz, CDCl_3_) δ_H_ 2.49 (t, *J* = 8.5 Hz, 4H, H-2′′, H-6′′), 2.69 (t, *J* = 6.0 Hz, 2H, H-2′), 3.20 (q, *J* = 5.5 Hz, 2H, H-1′), 3.74 (t, *J* = 5.0 Hz, 4H, H-3′′, H-5′′), 5.66 (s, 1H, H-3), 6.49 (bs, 1H, -NH), 7.12 (dd, *J* = 2.7, 6.8 Hz, 1H, H-8), 7.57–7.61 (m, 2H, H-6, H-7), 11.56 (s, 1H, 5-OH); ^13^C-NMR (125 MHz, CDCl_3_) δ_C_ 38.36 (C-1′), 53.16 (C-2′′, C-6′′), 55.49 (C-2′), 66.65 (C-3′′, C-5′′), 99.97 (C-2), 114.94 (C-9), 119.05 (C-6), 125.92 (C-8), 130.55 (C-10), 133.96 (C-7), 148.65 (C-2), 161.11 (C-5), 181.01 (C=O), 188.97 (C=O); LC-MS (ESI^−^, *m*/*z*) calculated for C_16_H_18_N_2_O_4_: 302.13, found for 301.16 [M − H]^−^; HRMS-ESI: *m*/*z* calculated for C_16_H_18_N_2_O_4_: 302.1267 [M]^+^, found for 302.1261.

*2-[(3-Aminopropyl)-morpholine]-5-hydroxy-1*,*4-naphthoquinone* (**19a**). The reaction produced **19a** in 29.2% as a red solid; mp 84.5–85.6 °C. ^1^H-NMR (500 MHz, CDCl_3_) δ_H_ 1.84 (p, *J* = 5.9 Hz, 2H, H-2′), 2.49 (bs, 4H, H-2′′, H-6′′), 2.54 (t, *J* = 5.8 Hz, 2H, H-3′), 3.27 (q, *J* = 5.7 Hz, 2H, H-1′), 3.84 (t, *J* = 4.6 Hz, 4H, H-3′′, H-5′′), 5.55 (s, 1H, H-3), 7.20 (dd, *J* = 0.8, 8.0 Hz, 1H, H-8), 7.43 (t, *J* = 7.9 Hz, 1H, H-7), 7.56 (dd, *J* = 0.9, 7.6 Hz, 1H, H-6), 8.08 (bs, 1H, -NH), 13.17 (s, 1H, 5-OH); ^13^C-NMR (125 MHz, CDCl_3_) δ_C_ 23.21 (C-2′), 43.15 (C-1′), 53.83 (C-2′′, C-6′′), 57.91 (C-3′), 66.76 (C-3′′, C-5′′), 99.04 (C-2), 115.12 (C-9), 118.89 (C-6), 125.70 (C-8), 130.69 (C-10), 133.70 (C-7), 149.46 (C-3), 161.06 (C-5), 181.21 (C=O), 188.84 (C=O); LC-MS (ESI^−^, *m*/*z*) calculated for C_17_H_20_N_2_O_4_: 316.14, found for 315.10 [M − H]^−^; HRMS-ESI: *m*/*z* calculated for C_17_H_20_O_4_N_2_: 316.1423 [M]^+^, found for 316.1428.

*2-[(3-Aminopropyl)-morpholine]-5-hydroxy-1*,*4-naphthoquinone* (**19b**). The reaction produced **19b** in 30.2% as a red solid; mp 101.6–102.7 °C. ^1^H-NMR (500 MHz, CDCl_3_) δ_H_ 1.84 (p, *J* = 6.0 Hz, 2H, H-2′), 2.50 (bs, 4H, H-2′′, H-6′′), 2.55 (t, *J* = 5.8 Hz, 2H, H-3′), 3.25 (q, *J* = 5.5 Hz, 2H, H-1′), 3.84 (t, *J* = 4.6 Hz, 4H, H-3′′, H-5′′), 5.63 (s, 1H, H-3), 7.10 (dd, *J* = 2.2, 7.3 Hz, 1H, H-8), 7.56–7.60 (m, 2H, H-6, H-7), 7.99 (bs, 1H, -NH), 11.63 (s, 1H, 5-OH); ^13^C-NMR (125 MHz, CDCl_3_) δ_C_ 23.15 (C-2′), 43.24 (C-1′), 53.83 (C-2′′, C-6′′), 58.05 (C-3′), 66.78 (C-3′′, C-5′′), 100.77 (C-3), 114.22 (C-9), 118.60 (C-6), 122.05 (C-8), 133.76 (C-10), 137.55 (C-7), 148.55 (C-2), 161.69 (C-5), 182.17 (C=O), 186.51 (C=O); LC-MS (ESI^−^, *m*/*z*) calculated for C_17_H_20_N_2_O_4_: 316.14, found for 315.11 [M − H]^−^; HRMS-ESI: *m*/*z* calculated for C_17_H_20_O_4_N_2_: 316.1423 [M]^+^, found for 316.1428.

### 3.5. Biological Activity

#### 3.5.1. Cell Lines and Cell Culture Conditions

All cells were obtained from the Bioresource Collection and Research Center (Hsinchu, Taiwan). Human colorectal adenocarcinoma cells (HT-29) and human promyelocytic leukemia cells (HL-60) cells were cultured in RPMI 1640 (HyClone, Logan, USA) medium, supplemented with 10% fetal bovine serum (FBS, Gibco, Life Technologies, New York, NY, USA) and antibiotics (100 μg/mL streptomycin and 100 units/mL penicillin, Gibco, Life Technologies). Human colorectal adenocarcinoma cells (SW480), human breast adenocarcinoma cells (MCF-7), human hepatocellular carcinoma cells (HepG2), and normal murine embryonic liver cells (BNL CL.2) were cultured in Dulbecco’s modified Eagle’s medium (DMEM, HyClone, Logan, USA), supplemented with 10% fetal bovine serum (FBS) and antibiotics. Human cancer cells were maintained in humidified atmosphere with 5% CO_2_ and 95% air at 37 °C in a carbon dioxide incubator (SANYO, CO_2_ incubator, Osaka, Japan).

#### 3.5.2. Cell Viability Assay

The cytotoxic effect of 34 1,4-naphthoquinone derivatives on five human cancer cell lines was measured by 3-(4,5-dimethylthiazol-2-yl)-2,5-diphenyl tetrazolium bromide (MTT, Bionovas Biotechnology Co., Ltd., Toronto, ON, Canada) as described by Mosmann [26]. The MTT assay depends on the mitochondrial enzyme reduction of tetrazolium dye to detect and determine cell viability. Briefly, the cancer cells or normal cells were plated at 5 × 10^3^–1 × 10^4^ cells into 96-well plates and HL-60 cells were plated at 1 × 10^5^ cells/mL into 12-well plates. After overnight growth, cells were treated with various concentrations of 1,4-naphthoquinone derivatives for 24 h, 48 h and 72 h. At the end of treatment, final concentration of 0.5 mg/mL MTT was added, and the cells were incubated for a further 1.5 h. The absorbance was recorded on an ELISA plate reader (SpectraMax 340PC^384^, Molecular Devices, Sunnyvale, CA, USA) at 540 nm. Cell viability was determined as the test compound concentration required to inhibit tumor cell proliferation by 50% (IC_50_) from the dose-response curves. All data are reported as average values from triplicate samples and the experiments were repeated at least three times.

#### 3.5.3. Nuclear Staining with Hoechst 33258

HT-29 cells were plate at 7 × 10^5^ cells per 6-cm dish. After overnight, cells were treated with 0.5–2.5 μM plumbagin (**1**) and **11a** for 48 h. The cells of each dish were stained with 5 mM Hoechst 33258 (Sigma-Aldrich, St. Louis, MO, USA). After the cells were incubated in a dark room for 5 min, the cells were washed with PBS and then were observed using a fluorescent microscope (Zeiss, Axio Observer A1, Göttingen, Germany).

#### 3.5.4. Cell Cycle Distribution Analysis

HT-29 cells were plated at a 1 × 10^6^ cells per 6-cm dish and cultured overnight. Then various concentration of 0.5–2.5 μM plumbagin (**1**) and **11a** were added for 48 h. The cells of each dish were harvested, then washed once with PBS and fixed with methanol at 4 °C. After 18 h, the cells of each dish were harvested, washed once with PBS. The cells were resuspended in 473 μL of PBS containing 40 μg/mL propidium iodide (PI) and 40 μg/mL RNase. Cells were incubated in a dark room for 30 min at room temperature then subjected to cell cycle analysis using a FACScan flow cytometer (Becton Dickinson, San Jose, CA, USA) and analyzed using the ModFit 3.0 software (Verity Software House, Topsham, ME, USA, 2008).

#### 3.5.5. Assessment of Apoptotic Analysis

HT-29 cells were plated at a concentration of 1 × 10^6^ cells per 6-cm dish. After overnight growth, cells were treated with 0.5–2.5 μM plumbagin (**1**) and **11a** for 48 h. The cells of each dish were harvested, then washed once with PBS. The cells were resuspended in 500 μL of PBS containing 4 μg/mL PI and 4 μg/mL Annexin-V-FITC (apoptosis detection kit; R & D Systems, Taipei, Taiwan). Cells were incubated in a dark room for 30 min at room temperature then subjected to cell cycle analysis using a FACScan flow cytometer (Becton Dickinson, San Jose, CA, USA). Annexin V-FITC and PI emission were detected in the FL1 and FL2 channels of the flow cytometer, respectively. Flow cytometer data showed three distinct populations of HT-29 cells. The normal healthy cells, early apoptotic, late apoptotic, and necrosis cells were represented by the Annexin V-negative/PI-negative population, Annexin V-positive/PI-negative, Annexin V-positive/PI-positive, and Annexin V-negative/PI-positive cells, respectively. The data were analyzed using a FACScan flow cytometer (Becton Dickinson, San Jose, CA, USA) requipped with ModFit 3.0 software (Verity Software House, Topsham, ME, USA).

#### 3.5.6. Statistical Analysis

The values shown are the mean ± SD of three independent experiments. Data are statistically evaluated by the Student’s test from SigmaPlot 11.0 (Systat Software Inc., San Jose, CA, USA) and shown as significantly different when * *p* < 0.05, ** *p* < 0.01 and *** *p* < 0.001.

## 4. Conclusions

In summary, we have synthesized a series of 2-position and 3-position lipophilic substituted lawsone and juglone derivatives and shown their enhanced their anti-cancer activity. Among these compounds, a lawsone derivative **11a** showed a superior cytotoxicity to all others. Furthermore, **11a** has better cytotoxicity against human colorectal adenocarcinoma HT-29 cells, over other cell lines, with an IC_50_ value of 1.99 ± 0.04 μM for 48 h. Cell cycle distribution and apoptotic analysis by flow cytometry indicated that **11a** was arrested cell cycle in S phase and induced apoptosis in HT-29. The Hoechst 33258 staining study showed the nuclear condensation of apoptotic with **11a** treatment. Furthermore, compound **11a** has markedly induced apoptotic morphological changes and nuclear DNA fragmentation. The mechanism of cell growth inhibition of the compound remains the target of future investigations.

## Supplementary Materials

Supplementary materials can be accessed at: http://www.mdpi.com/1420-3049/20/07/11994/s1.
