# Supplementary material for: Synthesis and Biological Evaluation of Lipophilic 1,4-Naphthoquinone Derivatives against Human Cancer Cell Lines
Source: Molecules. 2015 Jun 30;20(7):11994–2015. doi: 10.3390/molecules200711994 (PMC6331847; doi:10.3390/molecules200711994)
Supplement: Supplementary file 1 [file molecules-20-11994-s001.pdf]

Supplementary Materials

Materials and Methods

Biological Assay

Cell Culture

Normal murine embryonic liver BNL CL.2 cell lines were maintained in Dulbecco's modified Eagle's medium (DMEM, HyClone, Logan, UT, USA), supplemented with 10% fetal bovine serum (FBS) and 1% penicillin/streptomycin in CO_2 incubator (SANYO, CO_2 incubator, Osaka, Japan) with a humidified atmosphere of 95% air and 5% CO_2 at 37 °C.

Cell Cytotoxicity Assay Using MTT Assay

The effects of plumbagin (**1**) and compound **11a** on the cell viability were determined using 3-(4,5-dimethylthiazol-2-yl)-2,5-diphenyltetrazolium bromide (MTT, Bionovas Biotechnology Co., Ltd., Toronto, ON, Canada) assay. Briefly, murine embryonic liver cells BNL CL.2 (7×10^3 cells/well) were seeded in 96-well culture plates. After 24 h incubation to allow cell attachment, the cell were incubated with or without various concentrations of plumbagin (**1**) and compound **11a** for 48 h. Ten microliter of MTT (5 mg/mL) was then added to each well, and the plates were incubated for an additional 2.5 h at 37 °C. The formazan crystals formed by MTT metabolism were solubilized by 100 µL DMSO to each well. The absorbance at 540 nm was measured with a microplate ELISA reader (Molecular Devices spectramax 340 PC^{384}, Molecular Devices, Sunnyvale, CA, USA). Values represent the mean ± SD form at least two independent experiments.

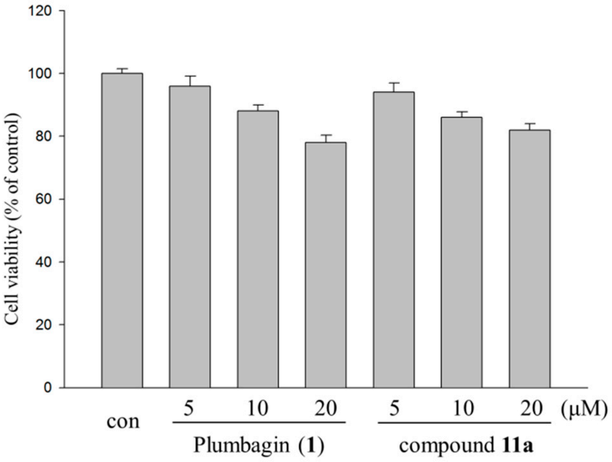

Figure S1. The effects of plumbagin (**1**) and compound **11a** on cell proliferative activity of normal cell line BNL CL.2 (murine embryonic liver cells) for 48 h.

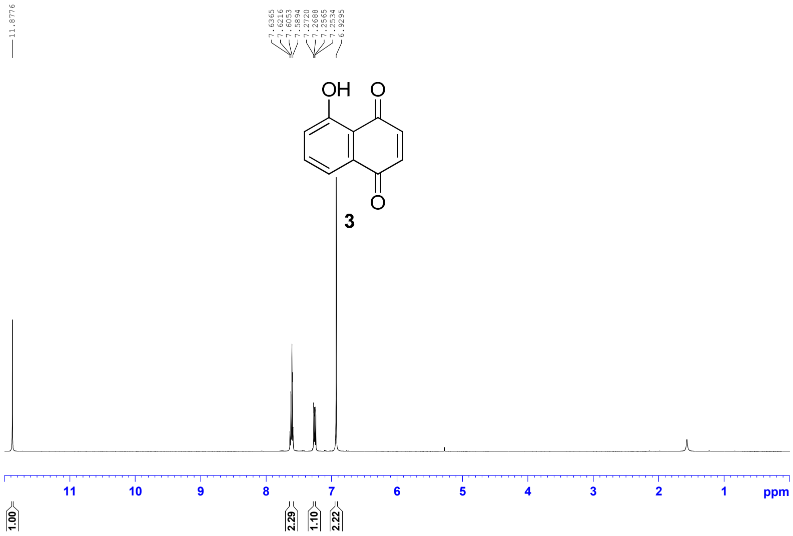

(A)

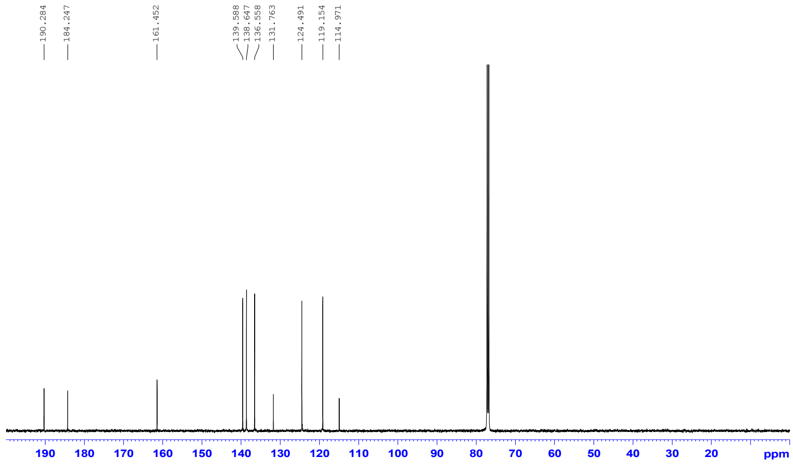

(B)

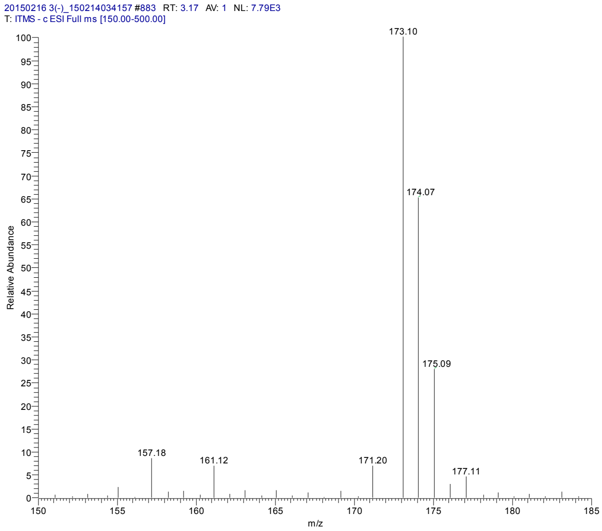

(C)

Figure S2. (**A**) ^{1}H-NMR spectra of juglone (**3**); (**B**) ^{13}C-NMR spectra of juglone (**3**); (**C**) LC-MS spectra of juglone (**3**).

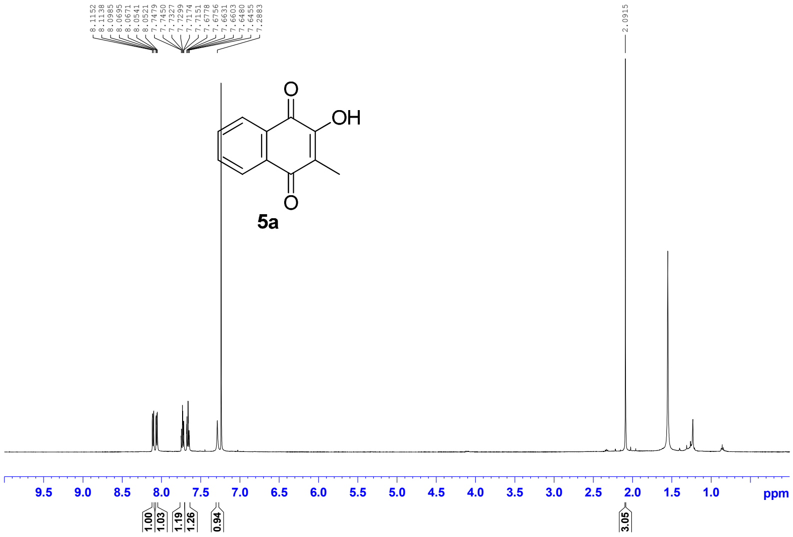

(A)

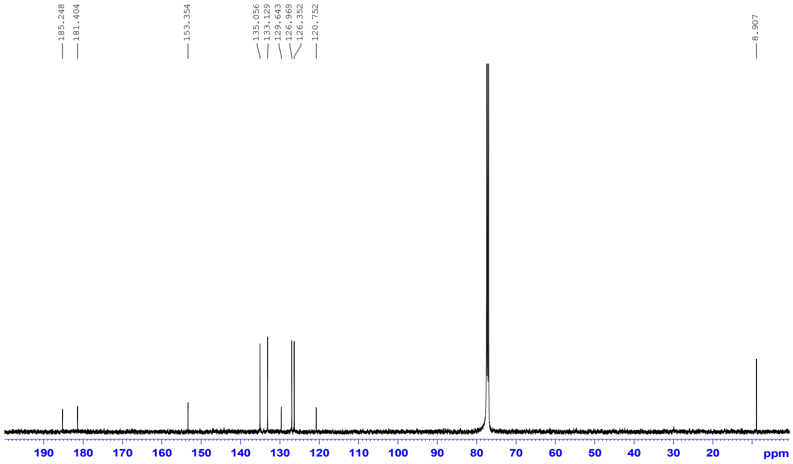

(B)

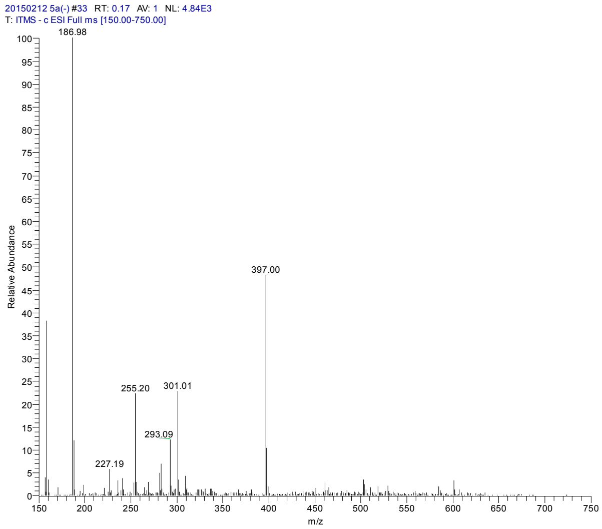

(C)

Figure S3. (**A**) ^{1}H-NMR spectra of compound **5a**; (**B**) ^{13}C-NMR spectra of compound **5a**; (**C**) LC-MS spectra of compound **5a**.

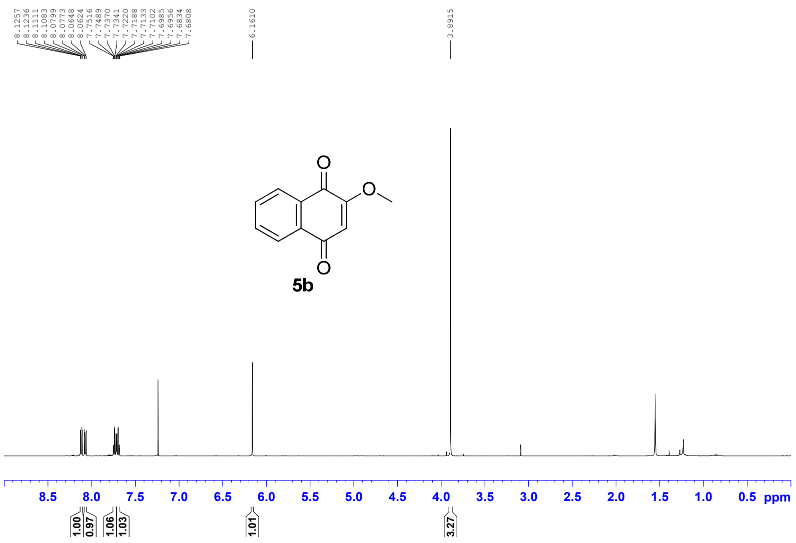

(A)

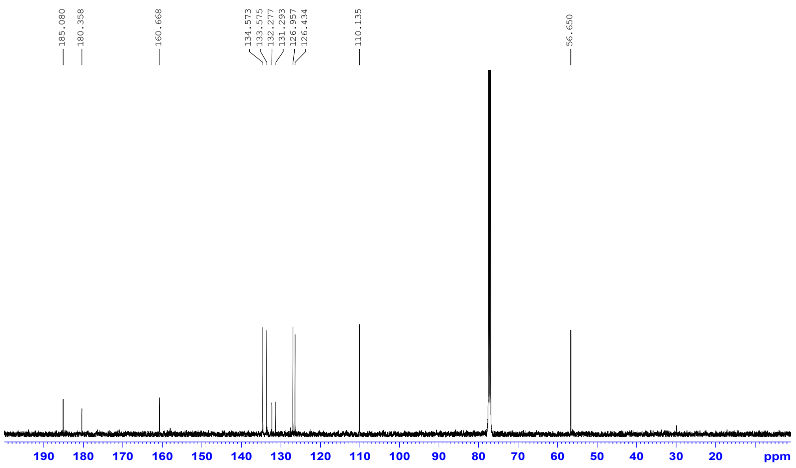

(B)

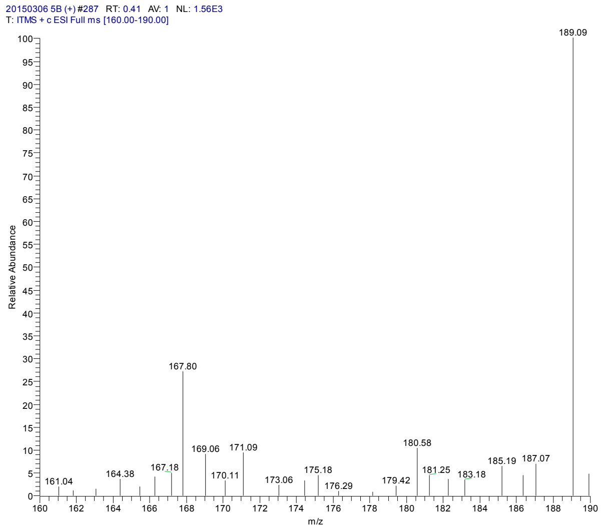

(C)

Figure S4. (**A**) ^{1}H-NMR spectra of compound **5b**; (**B**) ^{13}C-NMR spectra of compound **5b**; (**C**) LC-MS spectra of compound **5b**.

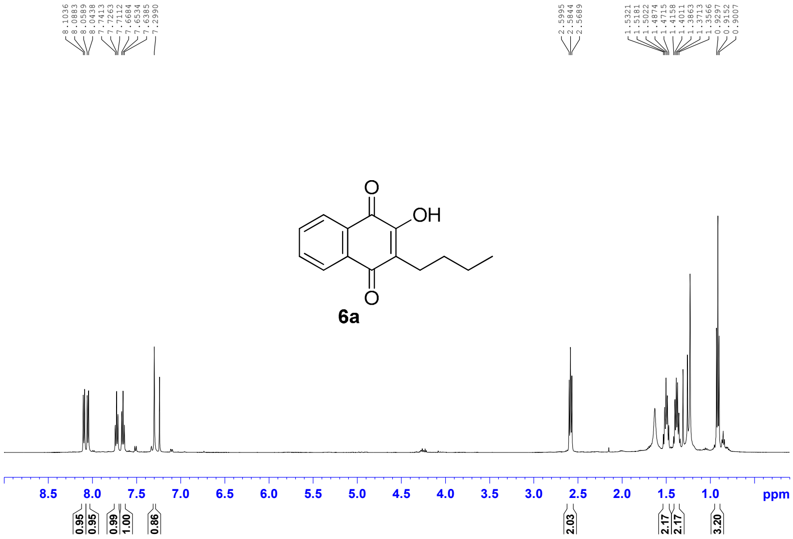

(**A**)

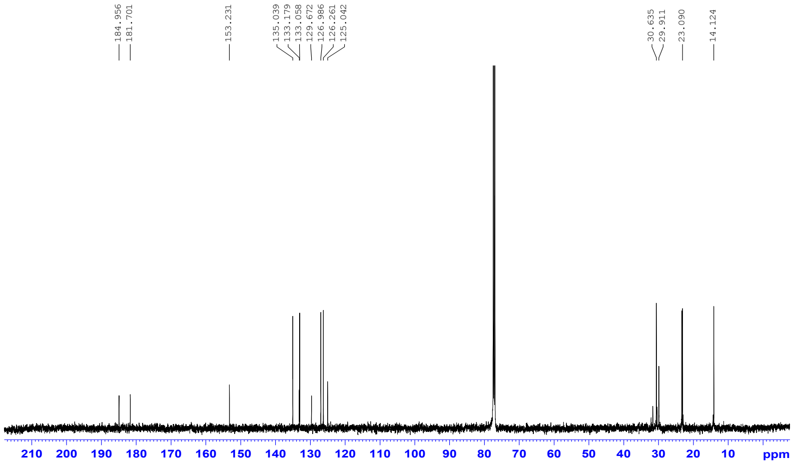

(**B**)

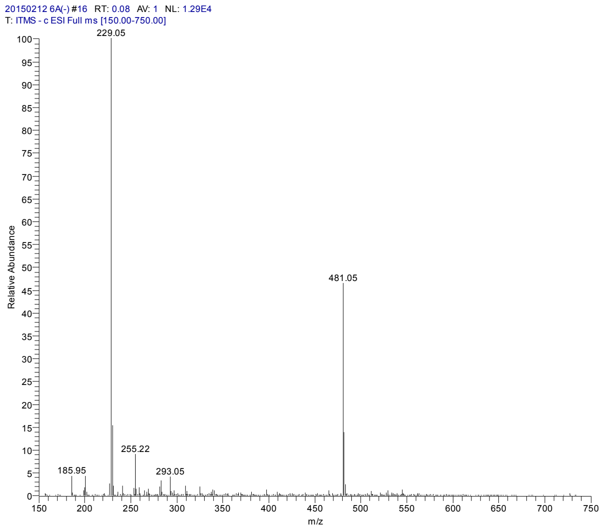

(**C**)

Figure S5. (**A**) ^{1}H-NMR spectra of compound **6a**; (**B**) ^{13}C-NMR spectra of compound **6a**; (**C**) LC-MS spectra of compound **6a**.

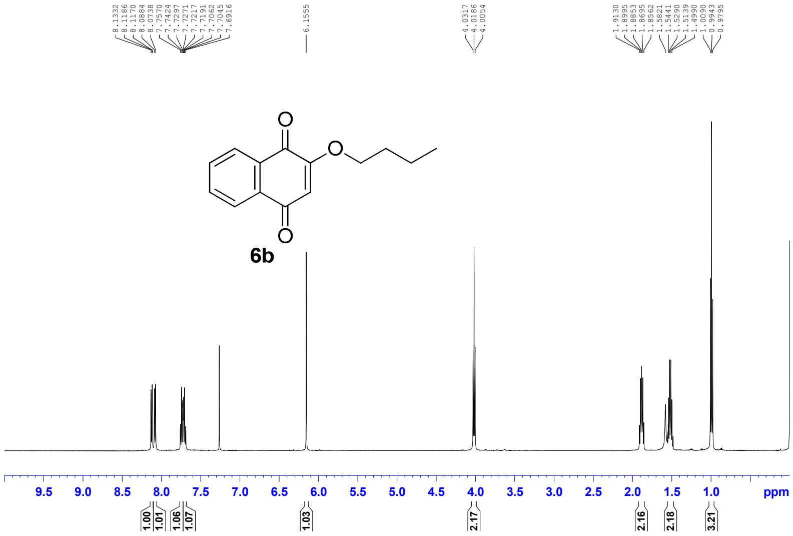

(**A**)

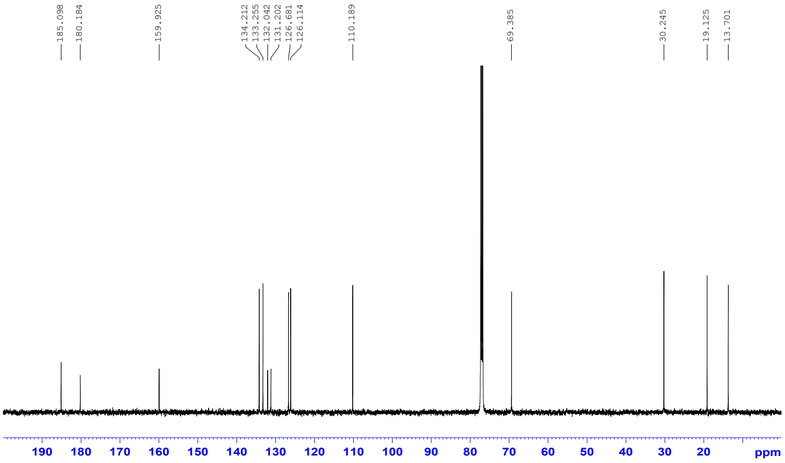

(**B**)

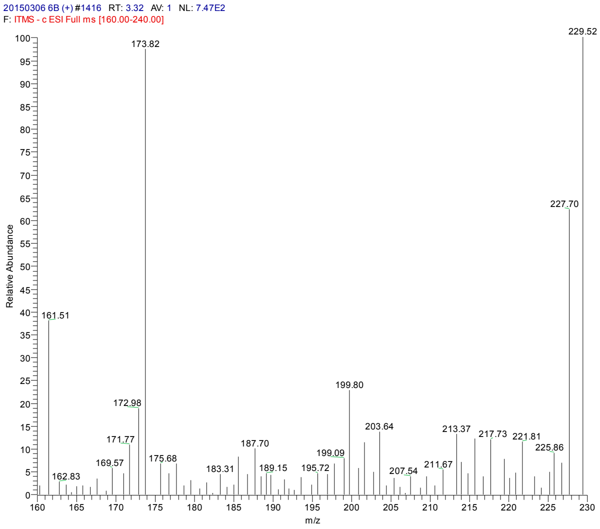

(**C**)

Figure S6. (**A**) ^{1}H-NMR spectra of compound **6b**; (**B**) ^{13}C-NMR spectra of compound **6b**; (**C**) LC-MS spectra of compound **6b**.

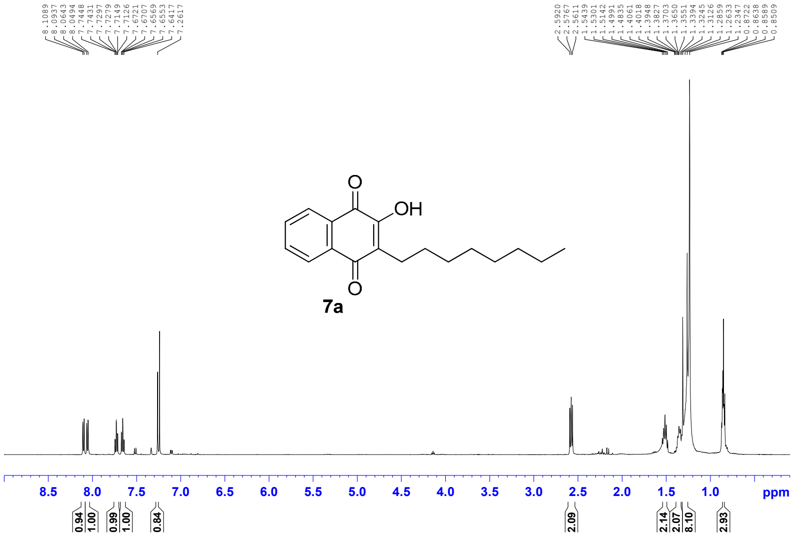

(A)

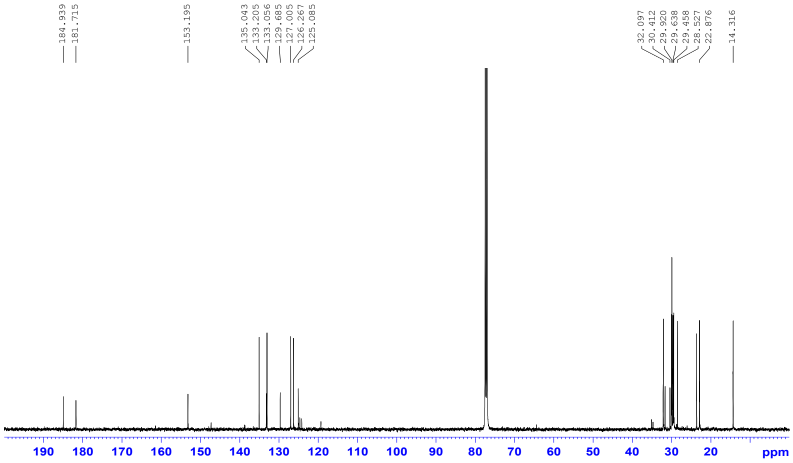

(B)

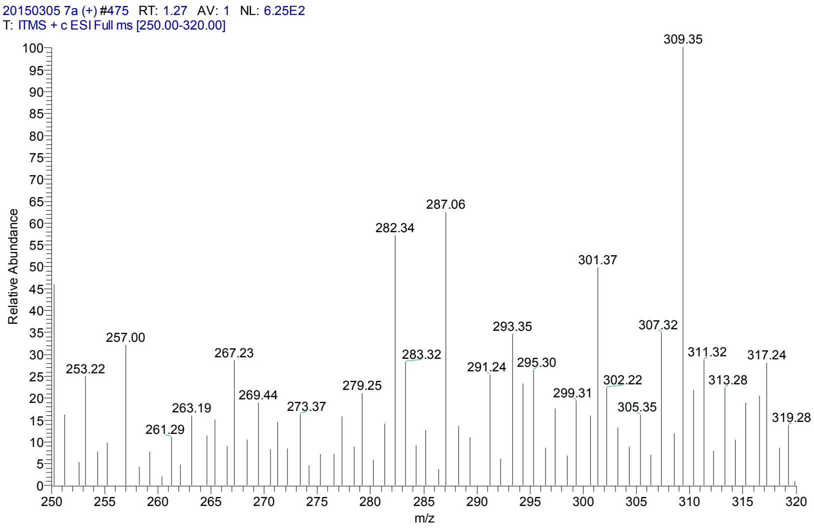

(C)

Figure S7. (**A**) ^{1}H-NMR spectra of compound **7a**; (**B**) ^{13}C-NMR spectra of compound **7a**; (**C**) LC-MS spectra of compound **7a**.

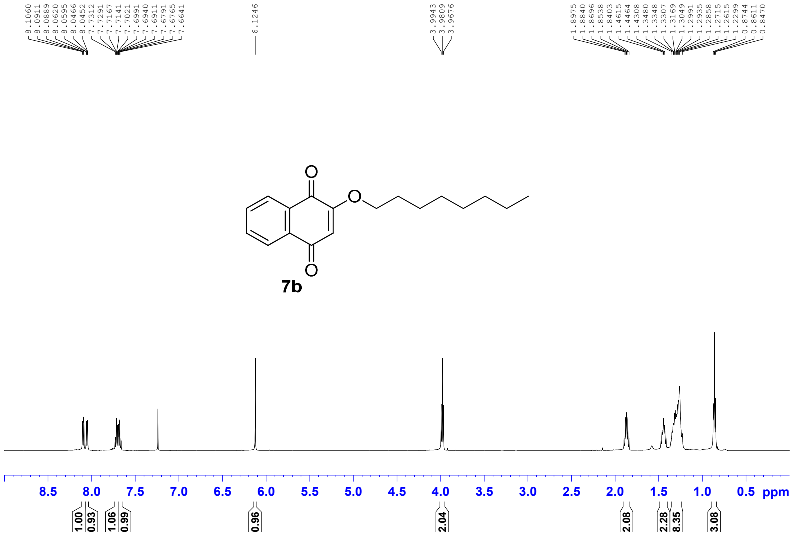

(A)

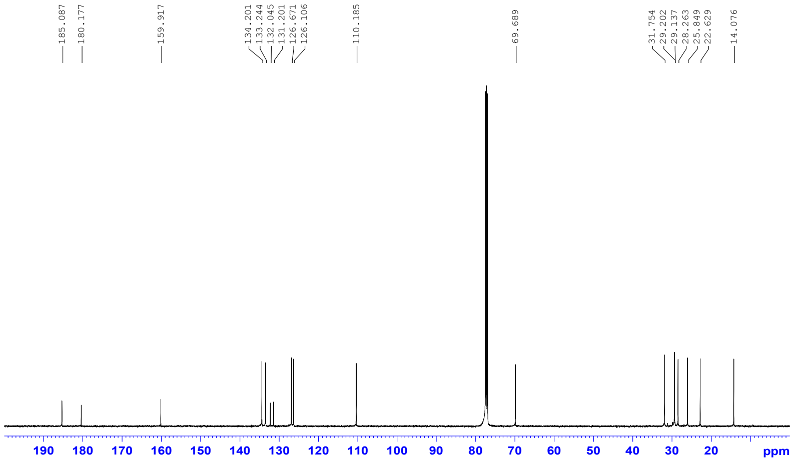

(B)

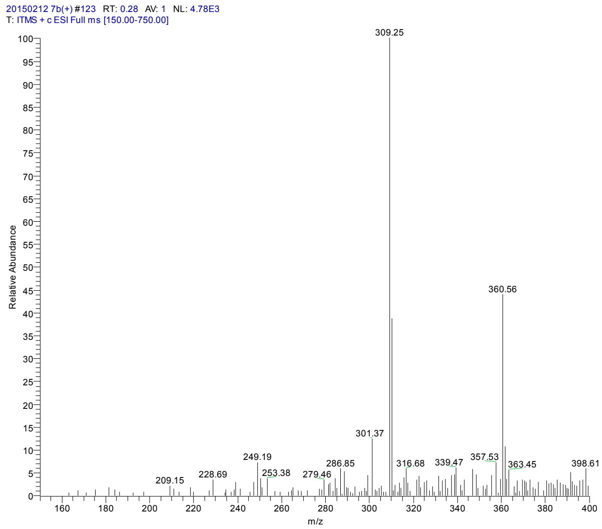

(C)

Figure S8. (**A**) ^{1}H-NMR spectra of compound **7b**; (**B**) ^{13}C-NMR spectra of compound **7b**; (**C**) LC-MS spectra of compound **7b**.

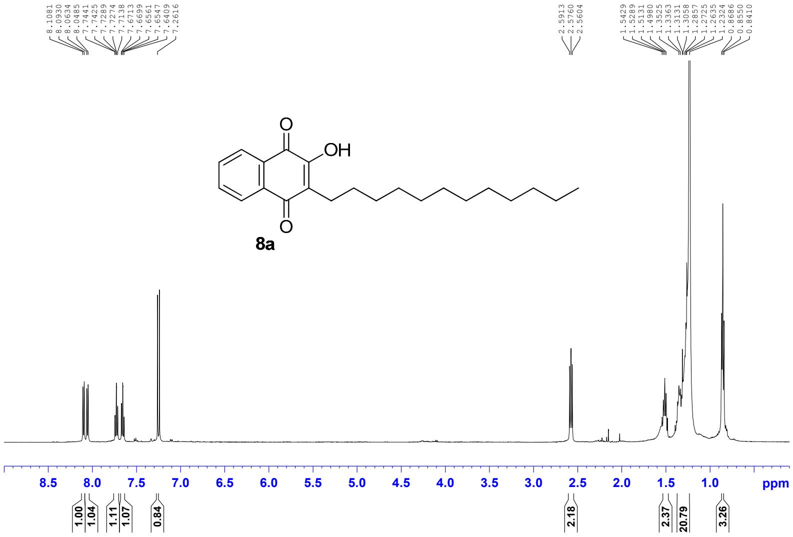

(**A**)

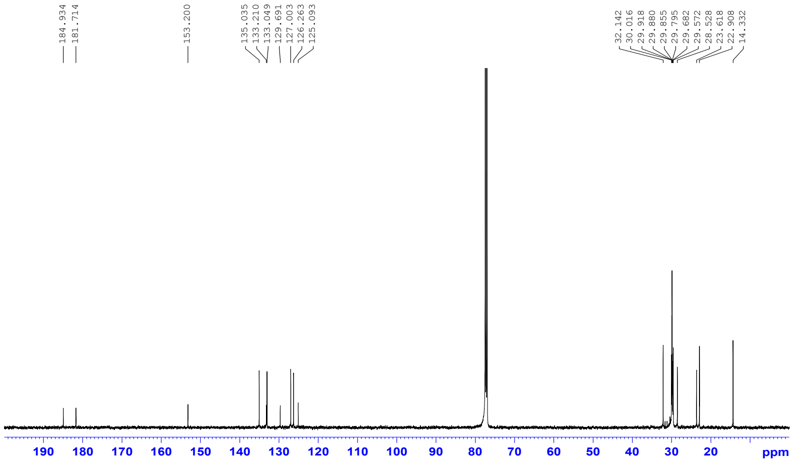

(**B**)

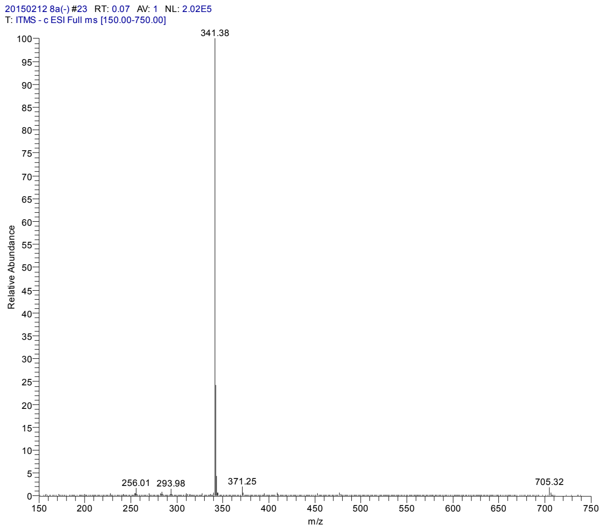

(**C**)

Figure S9. (**A**) ^{1}H-NMR spectra of compound **8a**; (**B**) ^{13}C-NMR spectra of compound **8a**; (**C**) LC-MS spectra of compound **8a**.

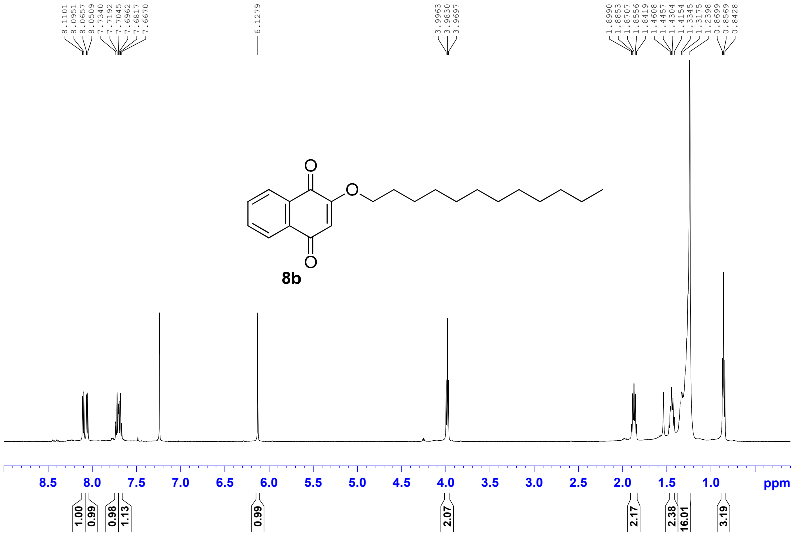

(A)

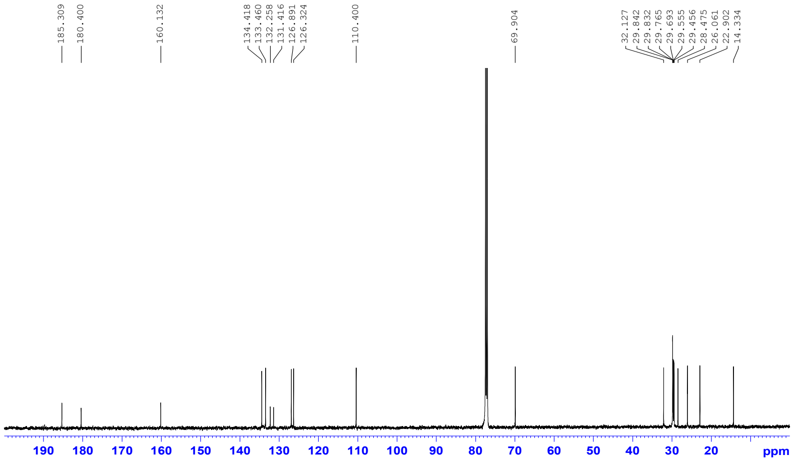

(B)

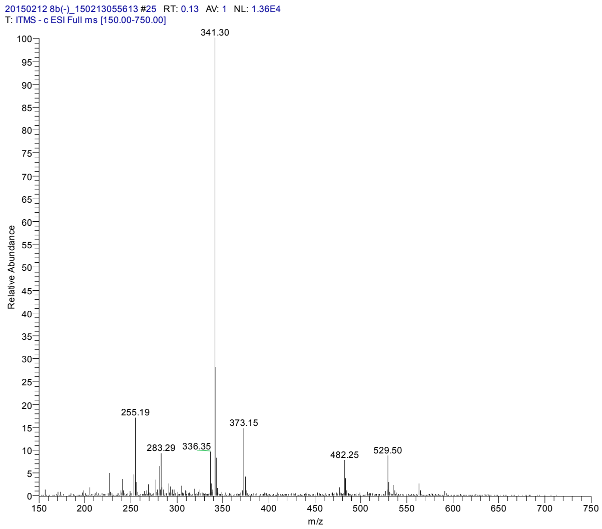

(C)

Figure S10. (**A**) ^{1}H-NMR spectra of compound **8b**; (**B**) ^{13}C-NMR spectra of compound **8b**; (**C**) LC-MS spectra of compound **8b**.

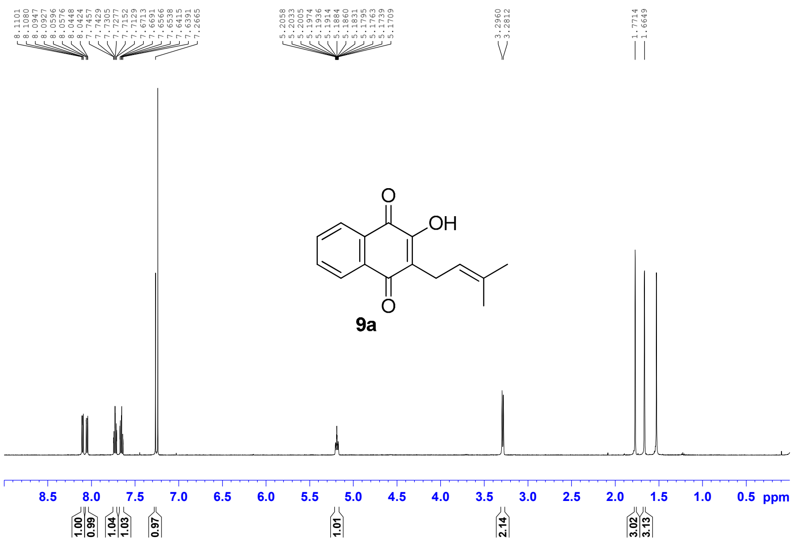

(A)

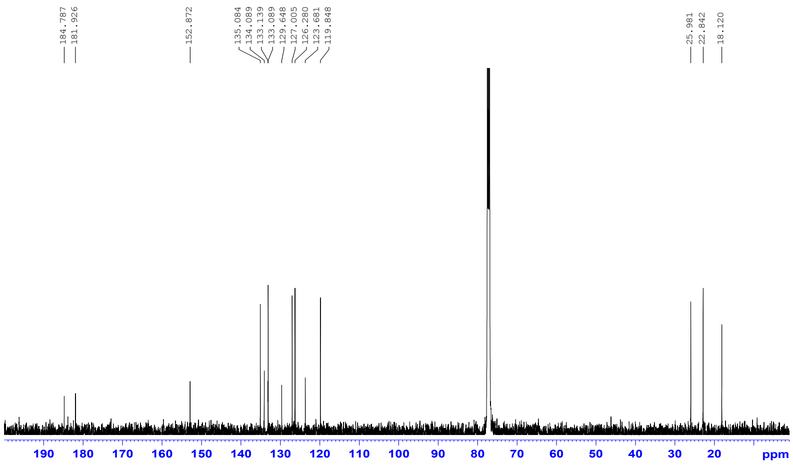

(B)

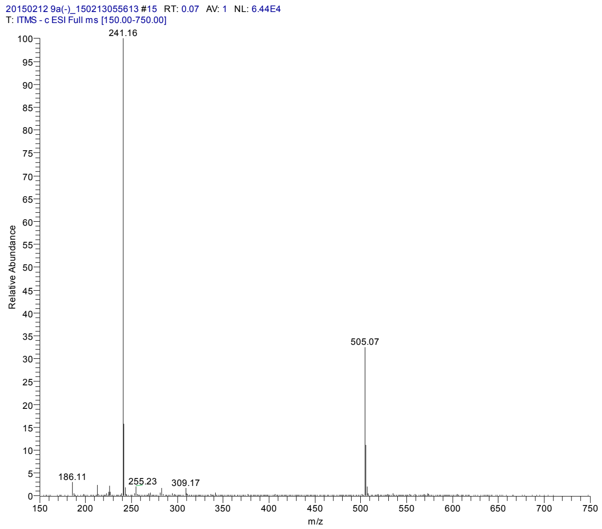

(C)

Figure S11. (**A**) ^{1}H-NMR spectra of compound **9a**; (**B**) ^{13}C-NMR spectra of compound **9a**; (**C**) LC-MS spectra of compound **9a**.

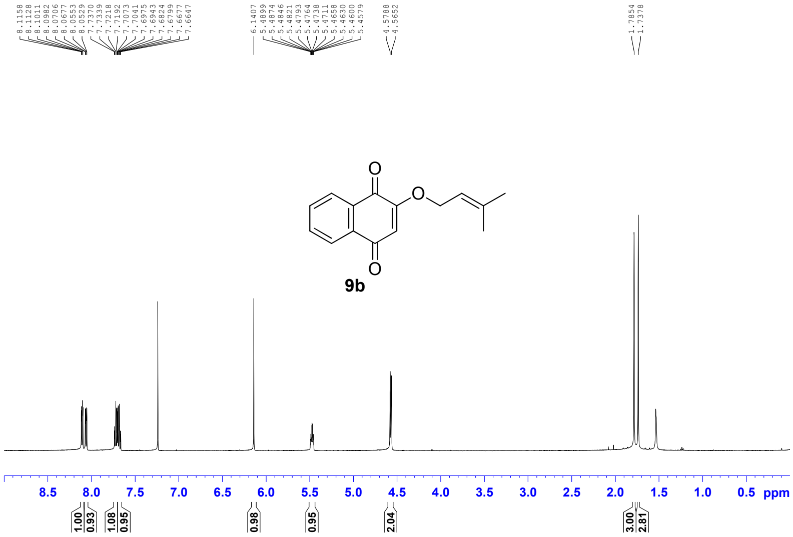

(A)

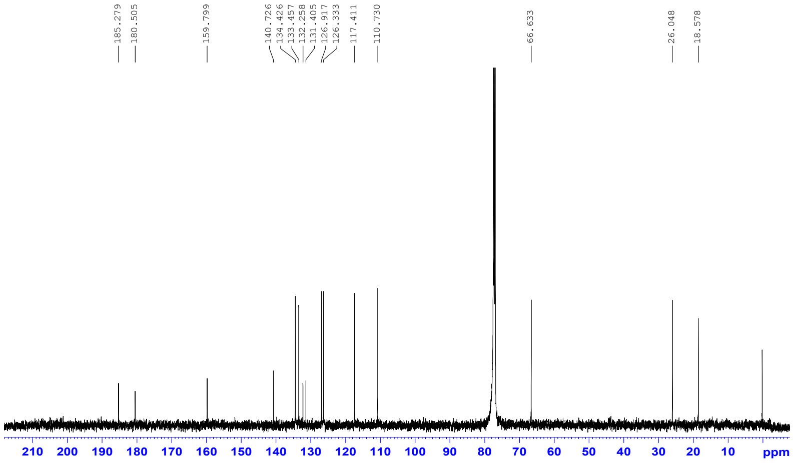

(B)

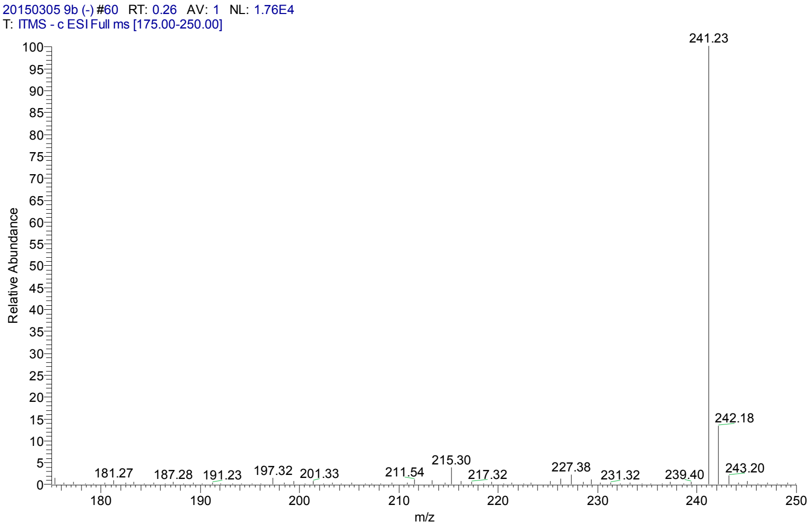

(C)

Figure S12. (**A**) ^{1}H-NMR spectra of compound **9b**; (**B**) ^{13}C-NMR spectra of compound **9b**; (**C**) LC-MS spectra of compound **9b**.

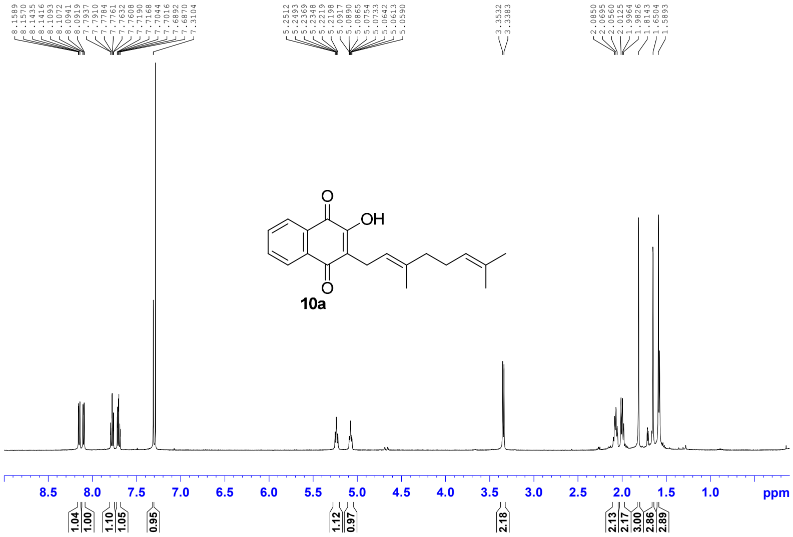

(A)

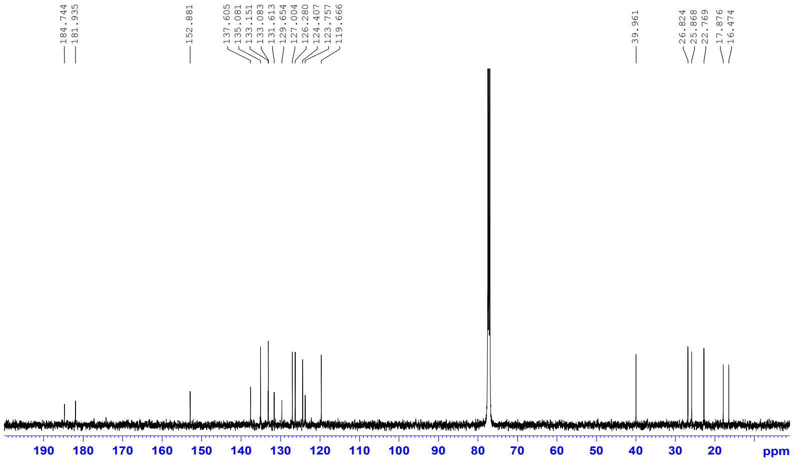

(B)

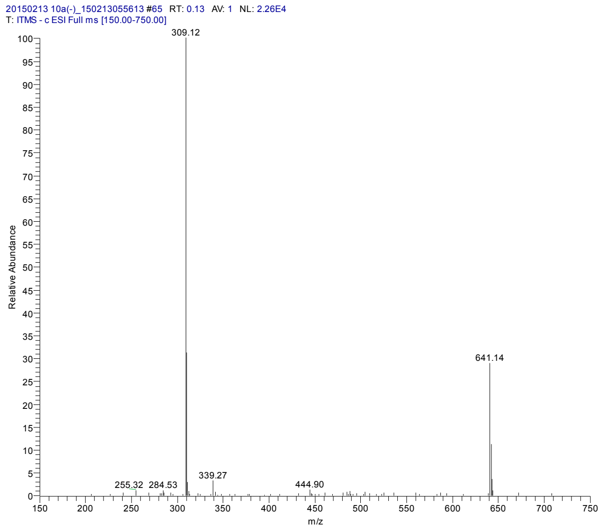

(C)

Figure S13. (**A**) ^{1}H-NMR spectra of compound **10a**; (**B**) ^{13}C-NMR spectra of compound **10a**; (**C**) LC-MS spectra of compound **10a**.

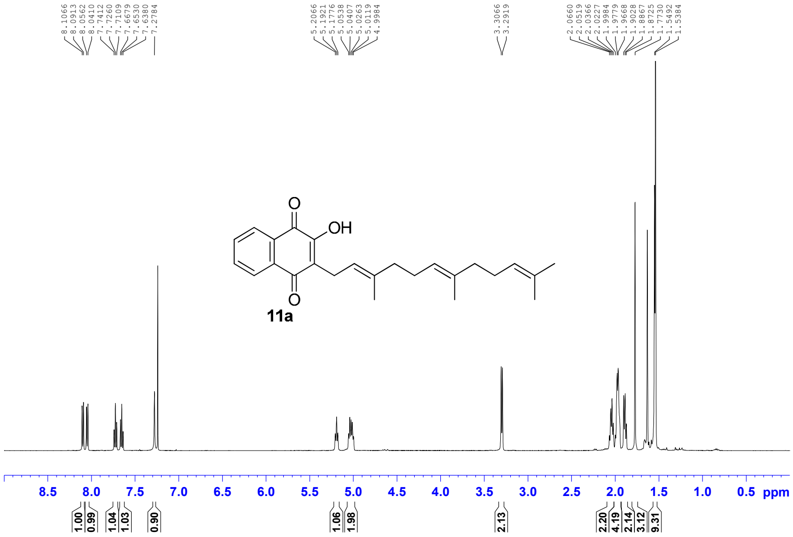

(A)

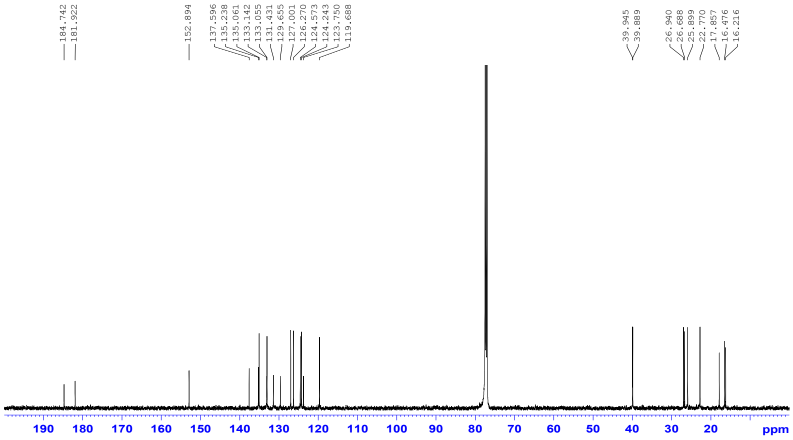

(B)

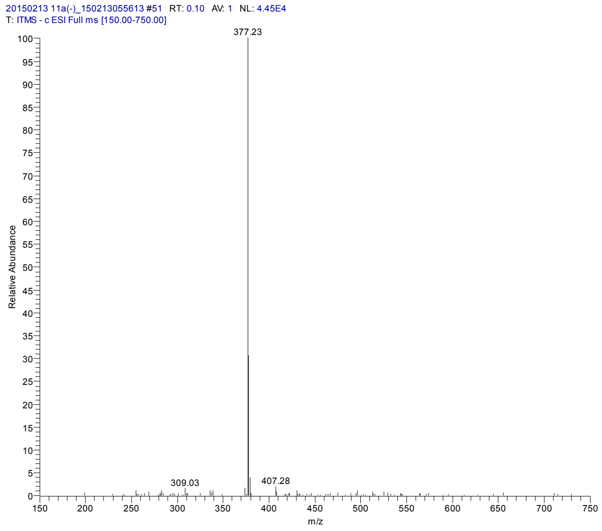

(C)

Figure S14. (**A**) ^{1}H-NMR spectra of compound **11a**; (**B**) ^{13}C-NMR spectra of compound **11a**; (**C**) LC-MS spectra of compound **11a**.

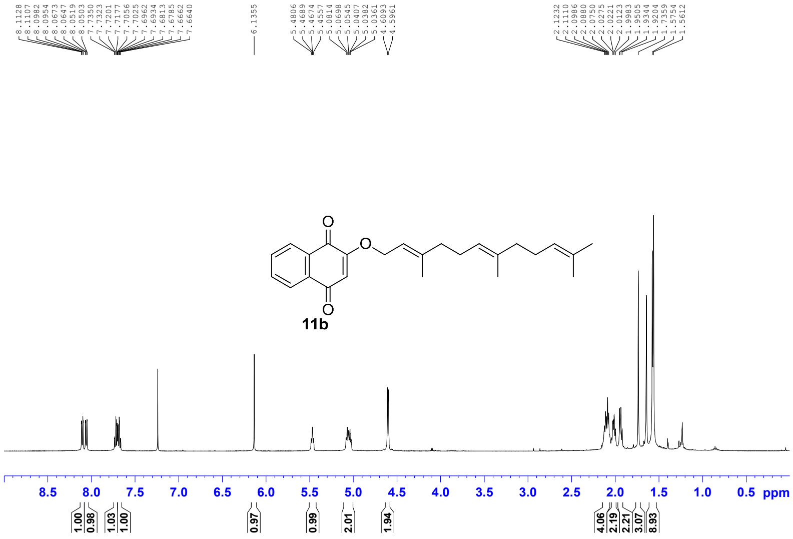

(A)

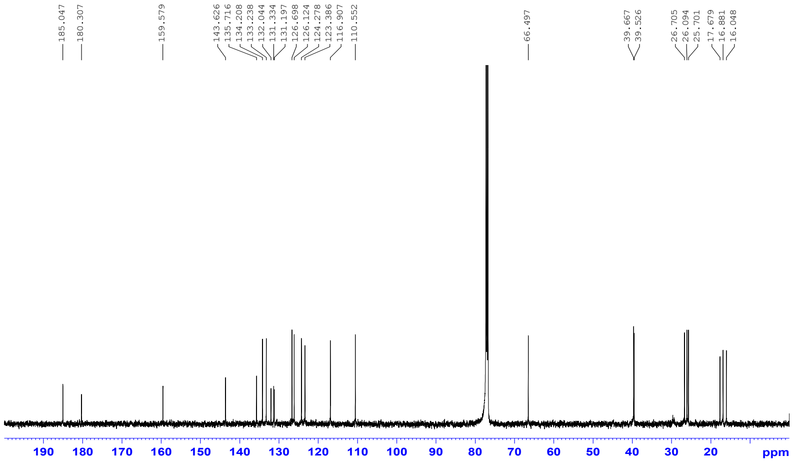

(B)

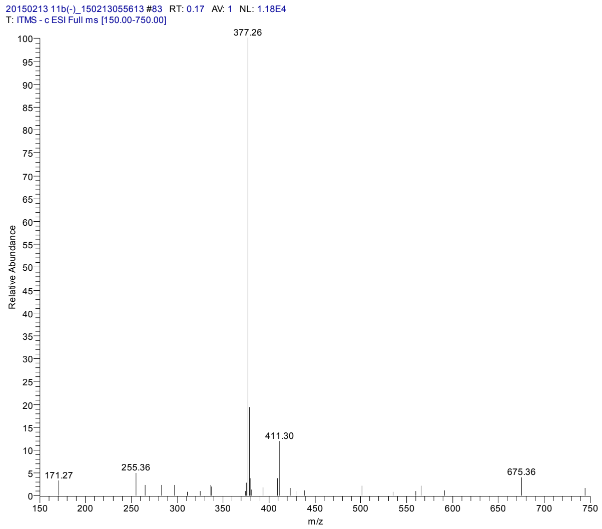

(C)

Figure S15. (**A**) ^{1}H-NMR spectra of compound **11b**; (**B**) ^{13}C-NMR spectra of compound **11b**; (**C**) LC-MS spectra of compound **11b**.

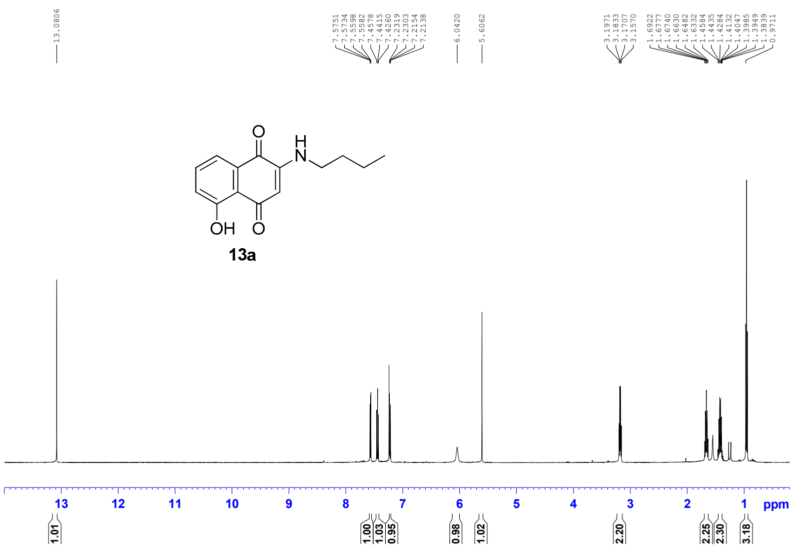

(**A**)

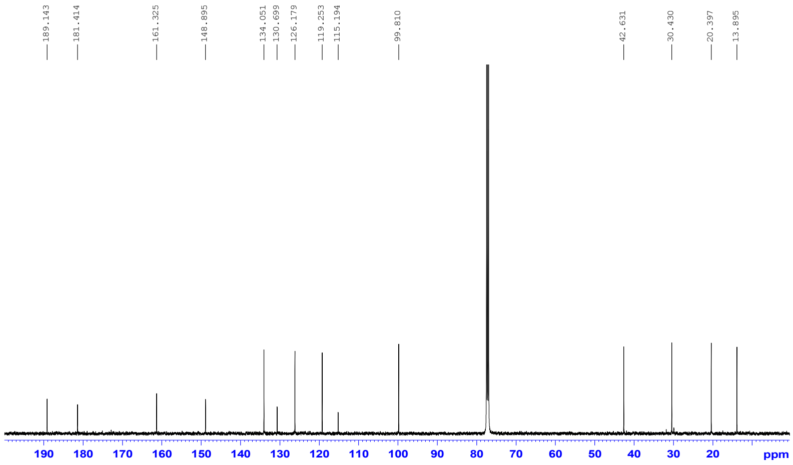

(**B**)

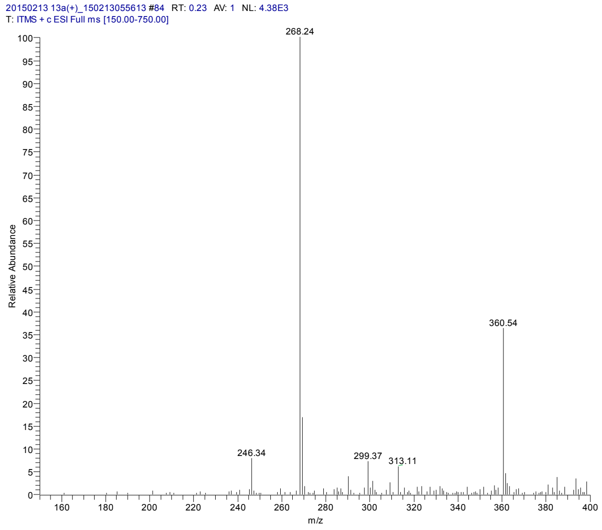

(**C**)

Figure S16. (**A**) ^{1}H-NMR spectra of compound **13a**; (**B**) ^{13}C-NMR spectra of compound **13a**; (**C**) LC-MS spectra of compound **13a**.

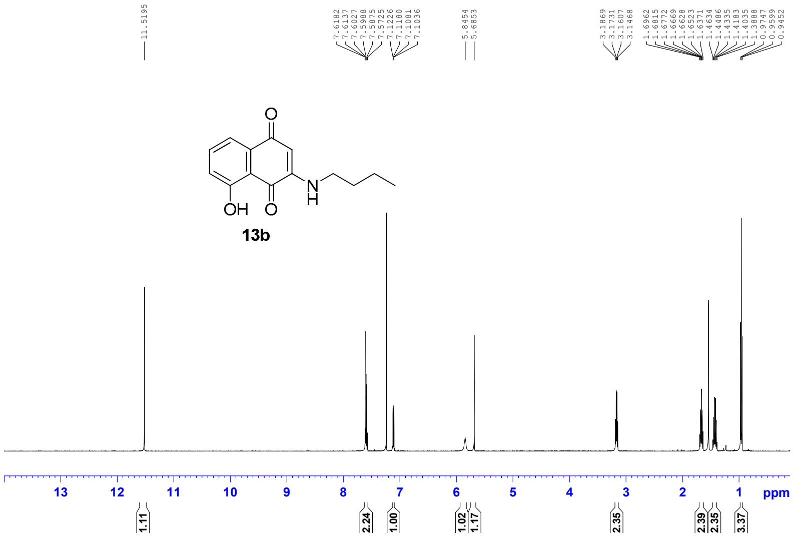

(A)

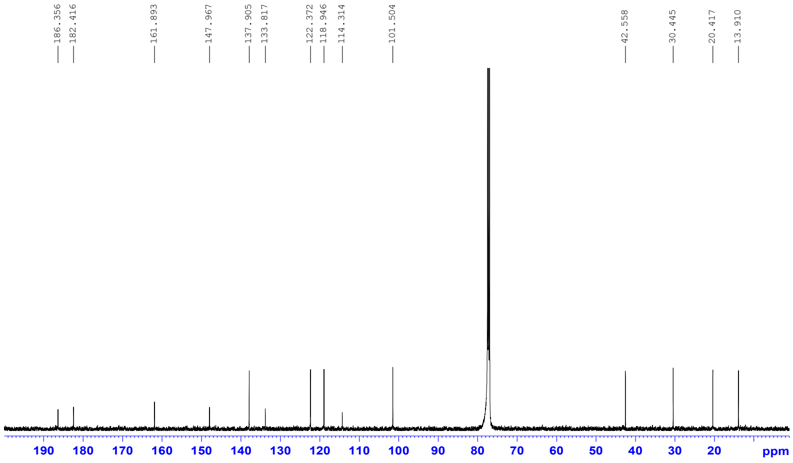

(B)

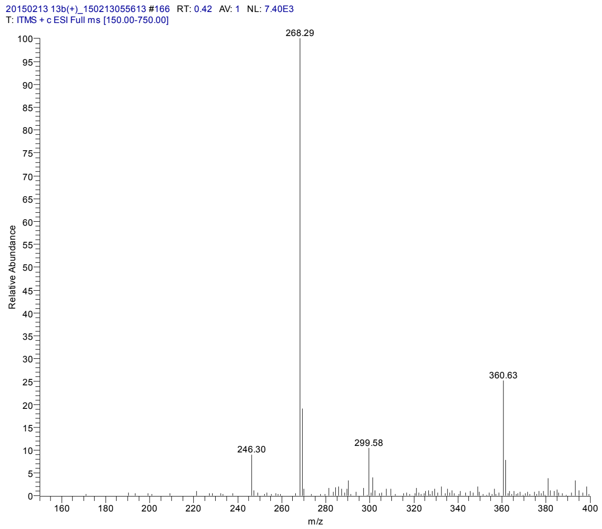

(C)

Figure S17. (**A**) ^{1}H-NMR spectra of compound **13b**; (**B**) ^{13}C-NMR spectra of compound **13b**; (**C**) LC-MS spectra of compound **13b**.

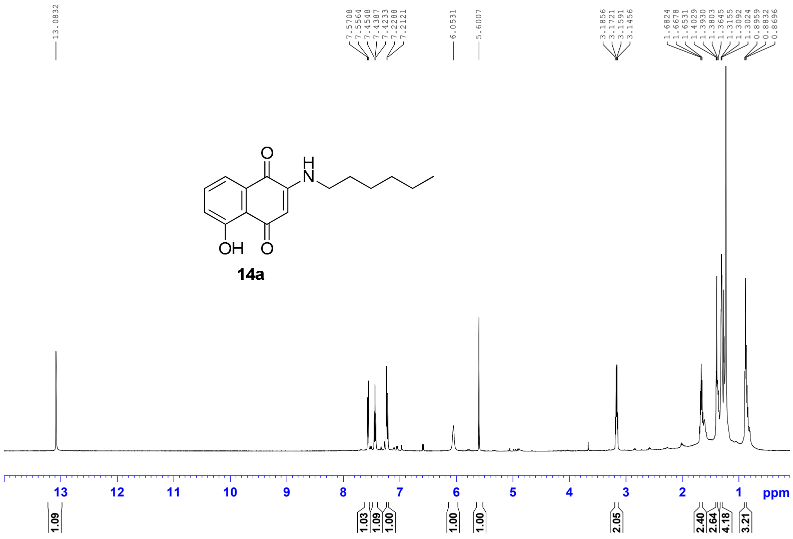

(**A**)

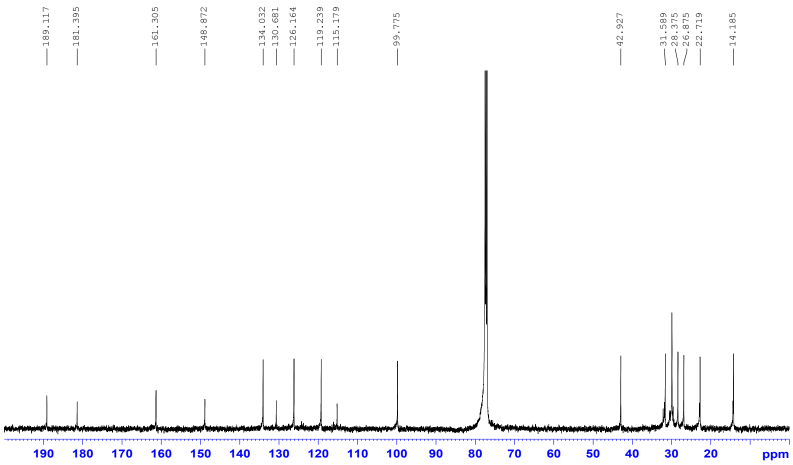

(**B**)

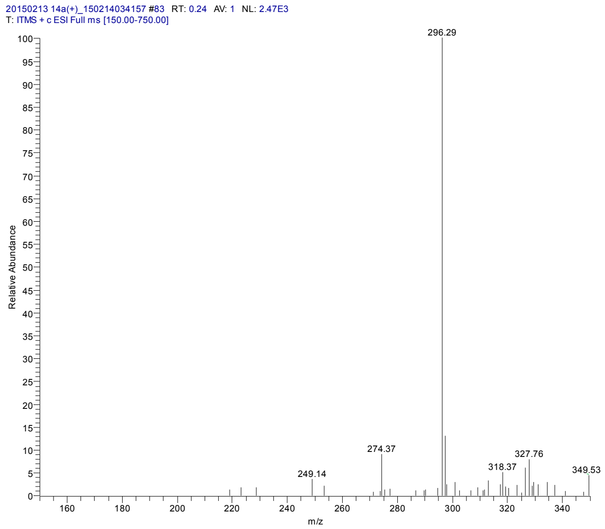

(**C**)

Figure S18. (**A**) ^{1}H-NMR spectra of compound **14a**; (**B**) ^{13}C-NMR spectra of compound **14a**; (**C**) LC-MS spectra of compound **14a**.

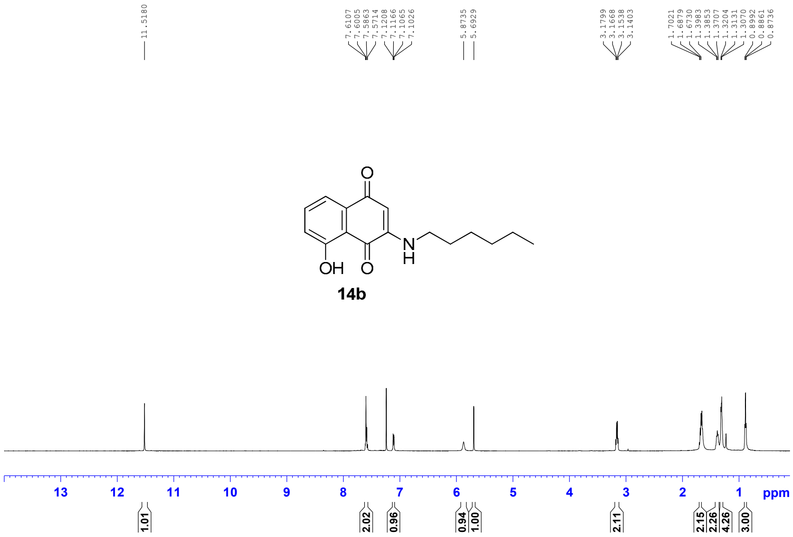

(**A**)

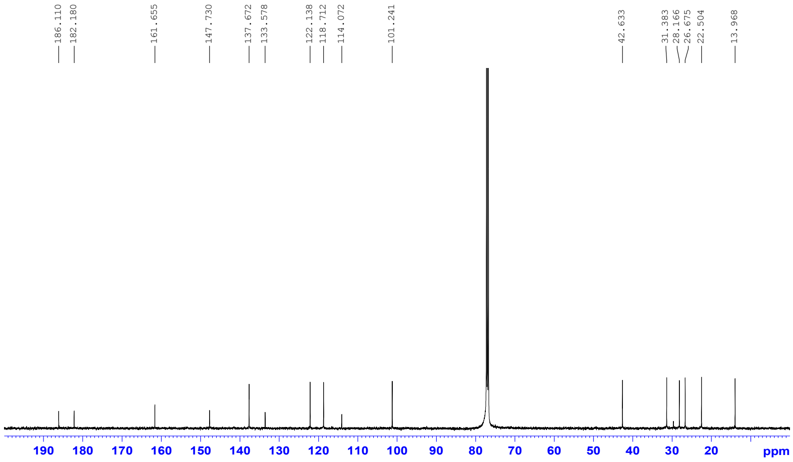

(**B**)

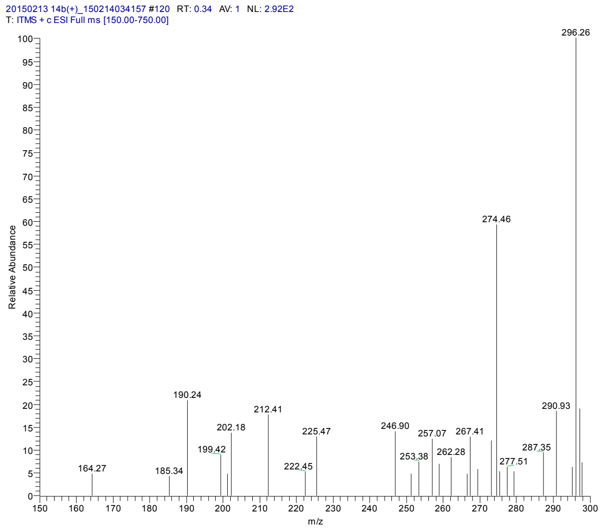

(**C**)

Figure S19. (**A**) ^{1}H-NMR spectra of compound **14b**; (**B**) ^{13}C-NMR spectra of compound **14b**; (**C**) LC-MS spectra of compound **14b**.

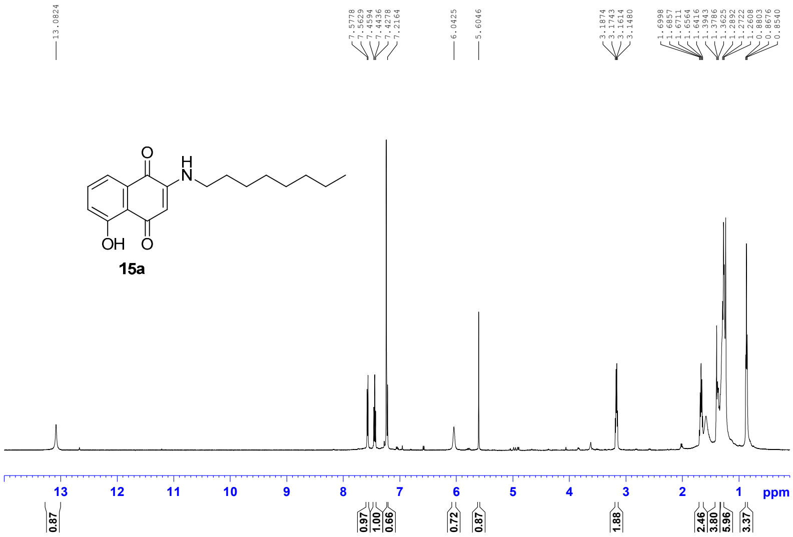

(A)

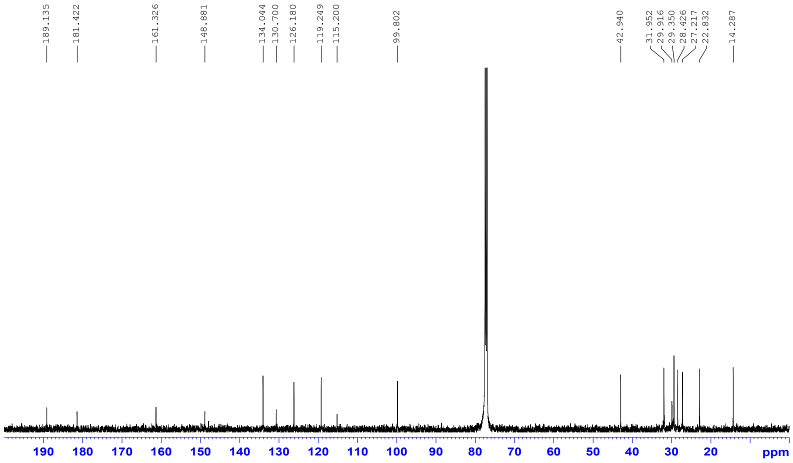

(B)

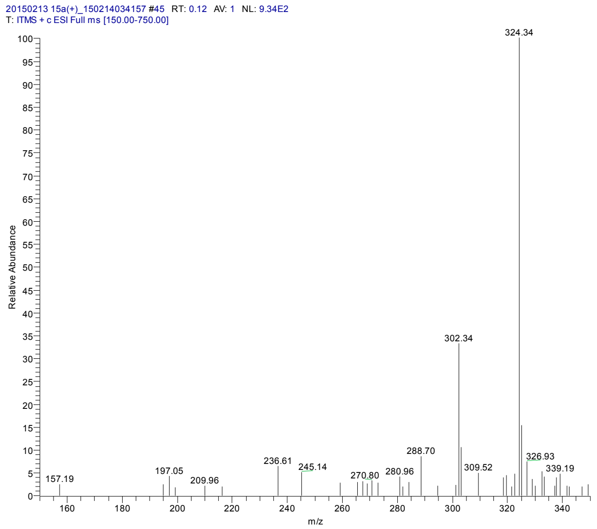

(C)

Figure S20. (**A**) ^{1}H-NMR spectra of compound **15a**; (**B**) ^{13}C-NMR spectra of compound **15a**; (**C**) LC-MS spectra of compound **15a**.

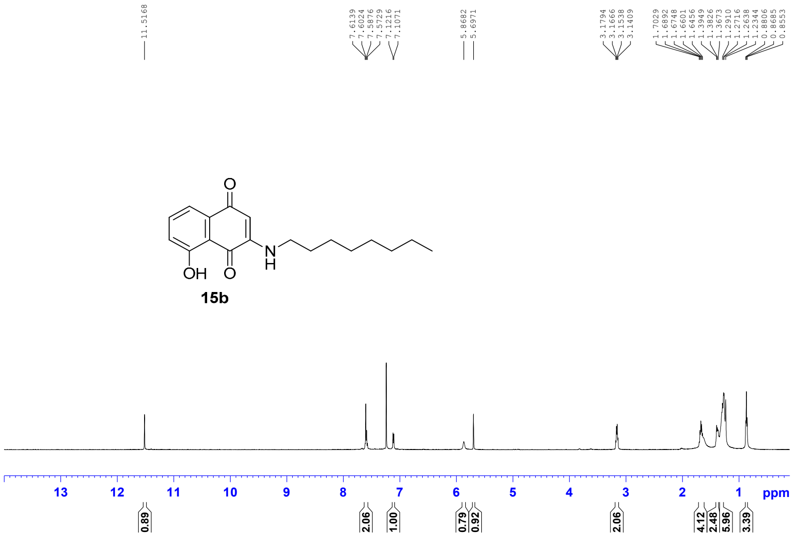

(A)

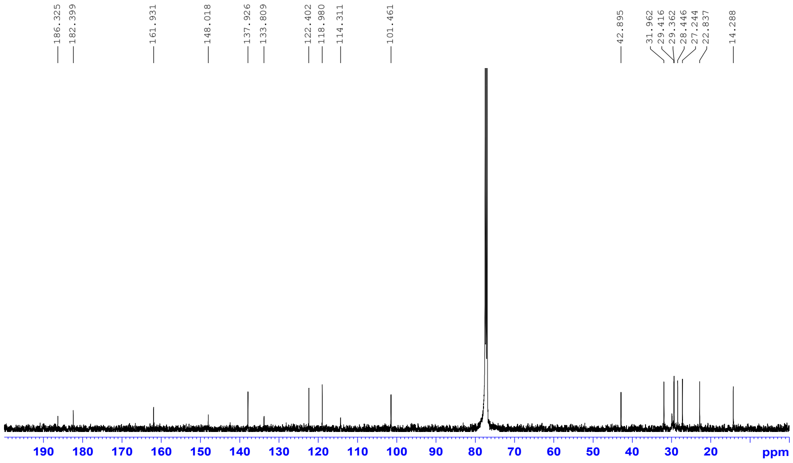

(B)

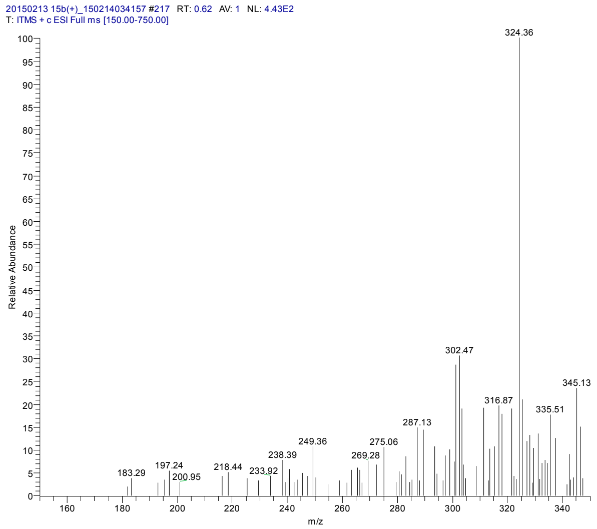

(C)

Figure S21. (**A**) ^{1}H-NMR spectra of compound **15b**; (**B**) ^{13}C-NMR spectra of compound **15b**; (**C**) LC-MS spectra of compound **15b**.

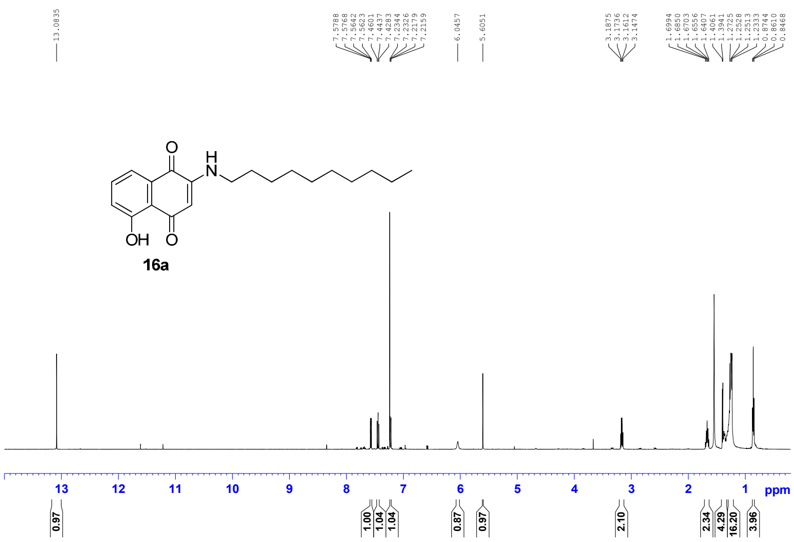

(A)

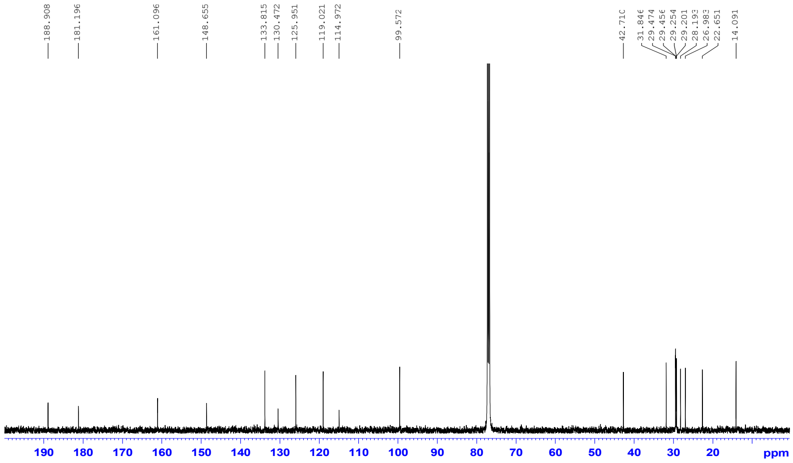

(B)

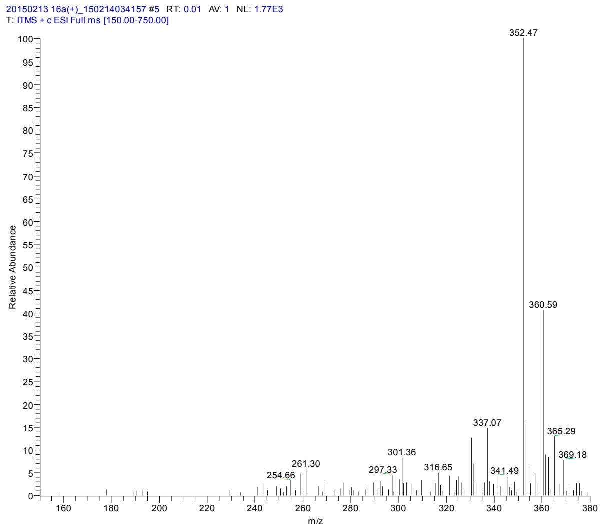

(C)

Figure S22. (**A**) ^{1}H-NMR spectra of compound **16a**; (**B**) ^{13}C-NMR spectra of compound **16a**; (**C**) LC-MS spectra of compound **16a**.

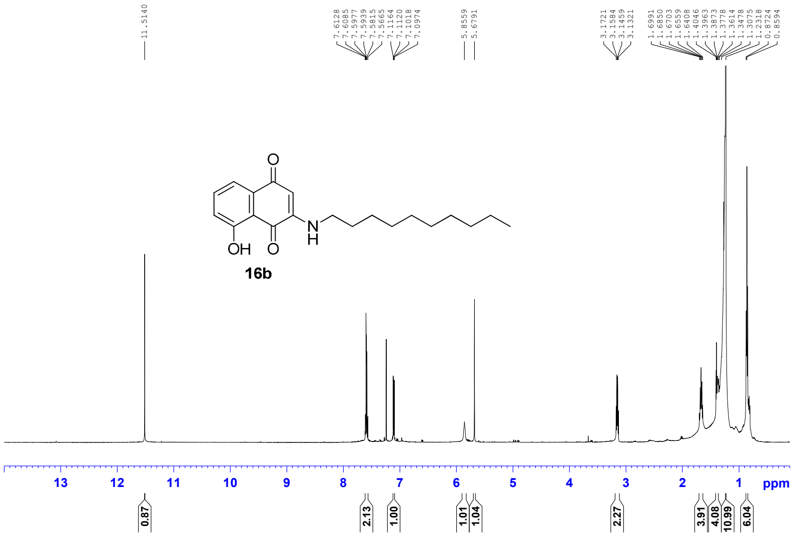

(**A**)

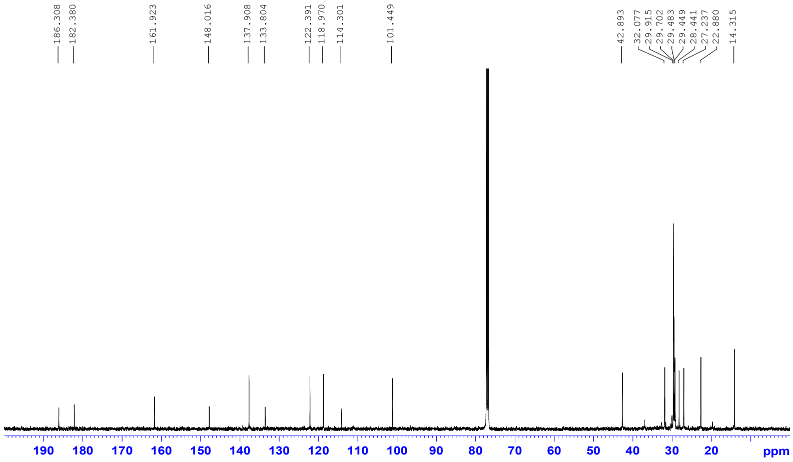

(**B**)

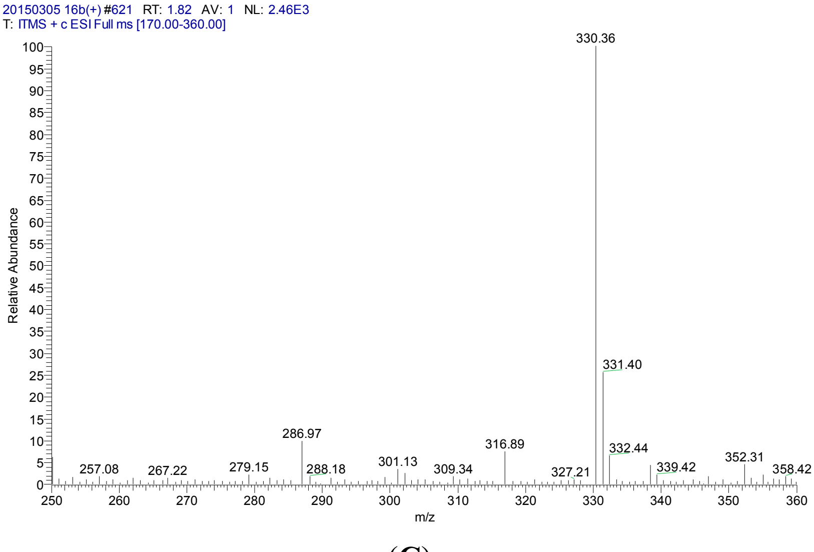

(**C**)

Figure S23. (**A**) ^{1}H-NMR spectra of compound **16b**; (**B**) ^{13}C-NMR spectra of compound **16b**; (**C**) LC-MS spectra of compound **16b**.

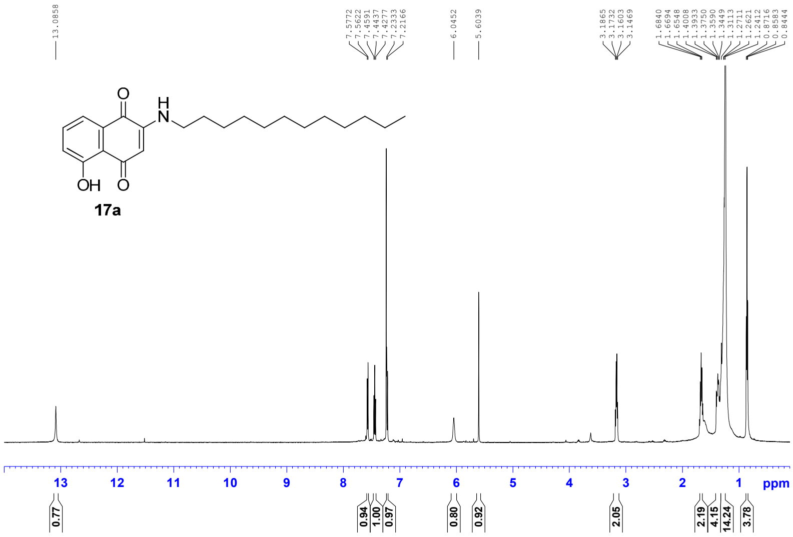

(A)

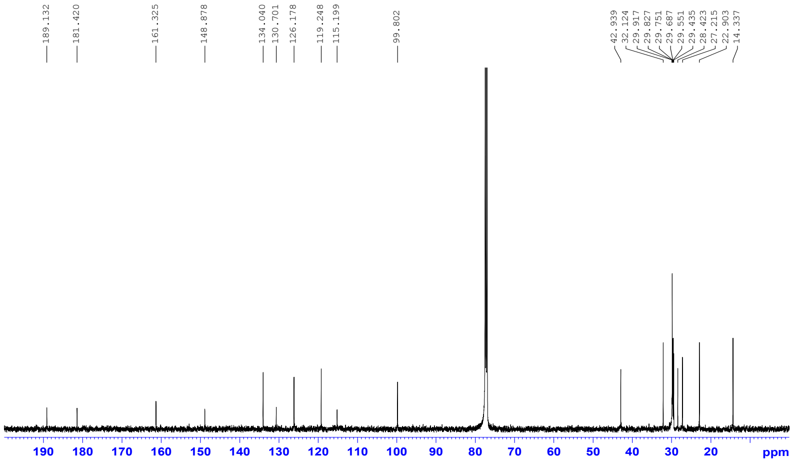

(B)

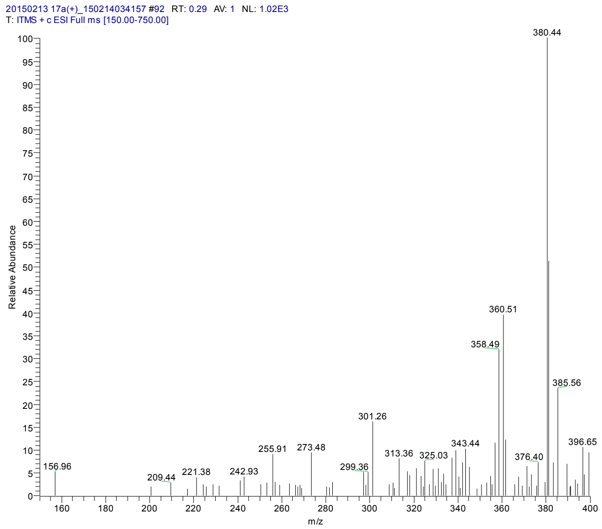

(C)

Figure S24. (**A**) ^{1}H-NMR spectra of compound **17a**; (**B**) ^{13}C-NMR spectra of compound **17a**; (**C**) LC-MS spectra of compound **17a**.

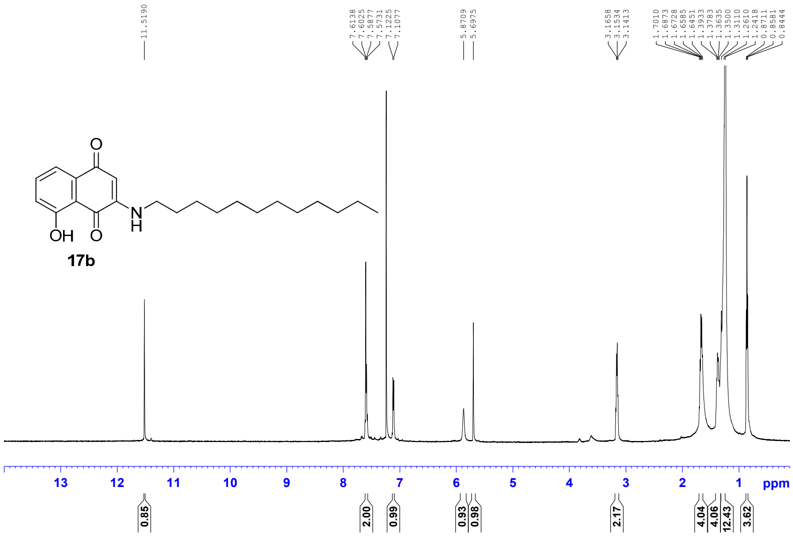

(**A**)

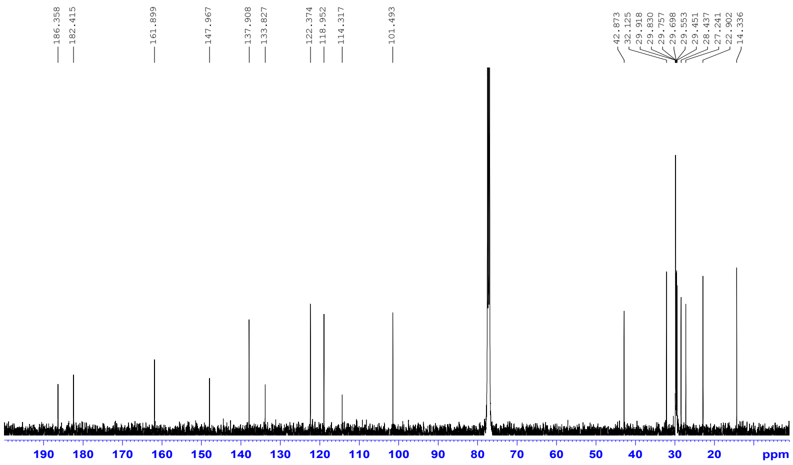

(**B**)

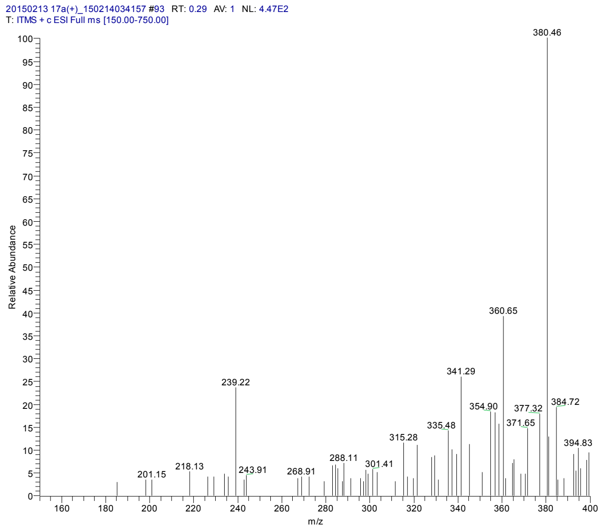

(**C**)

Figure S25. (**A**) ^{1}H-NMR spectra of compound **17b**; (**B**) ^{13}C-NMR spectra of compound **17b**; (**C**) LC-MS spectra of compound **17b**.

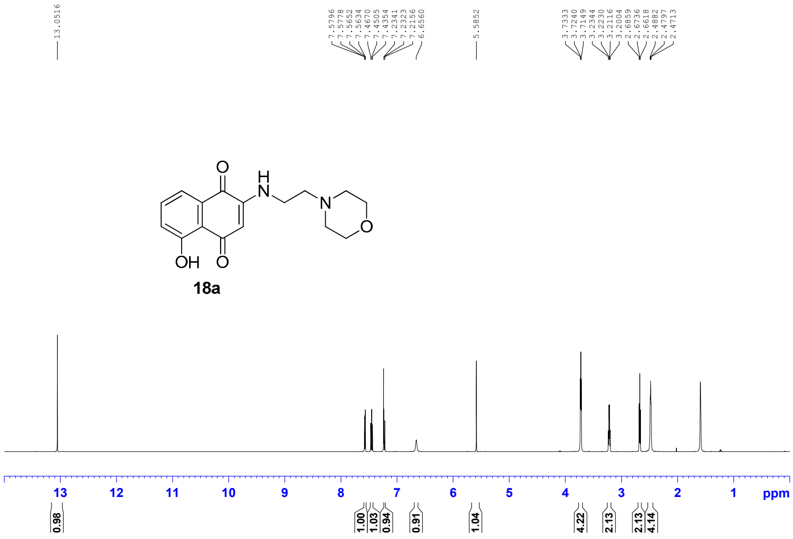

(A)

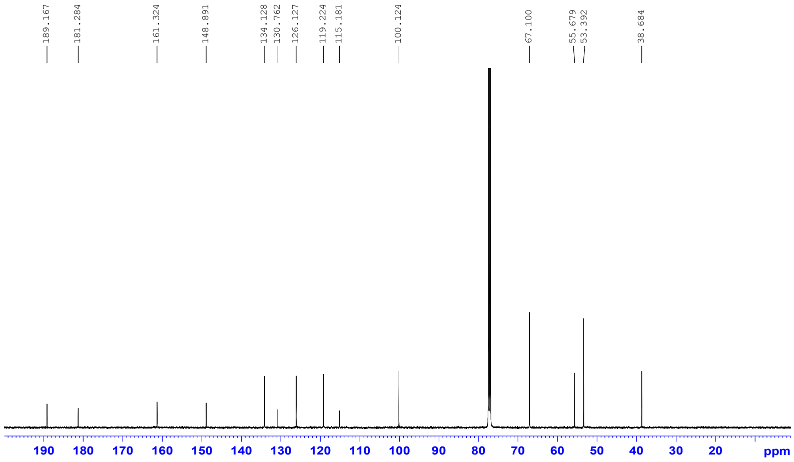

(B)

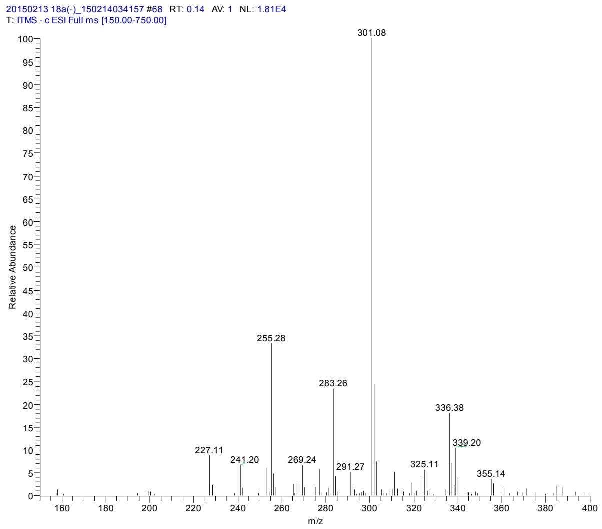

(C)

Figure S26. (**A**) ^{1}H-NMR spectra of compound **18a**; (**B**) ^{13}C-NMR spectra of compound **18a**; (**C**) LC-MS spectra of compound **18a**.

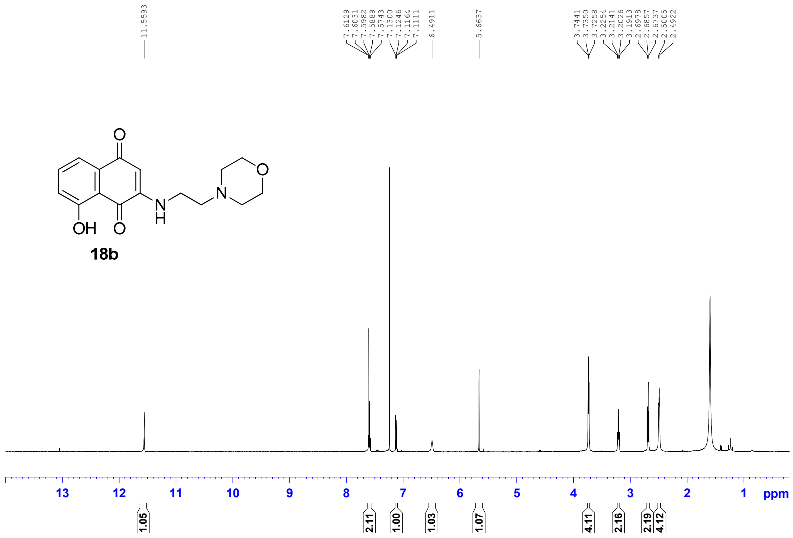

(**A**)

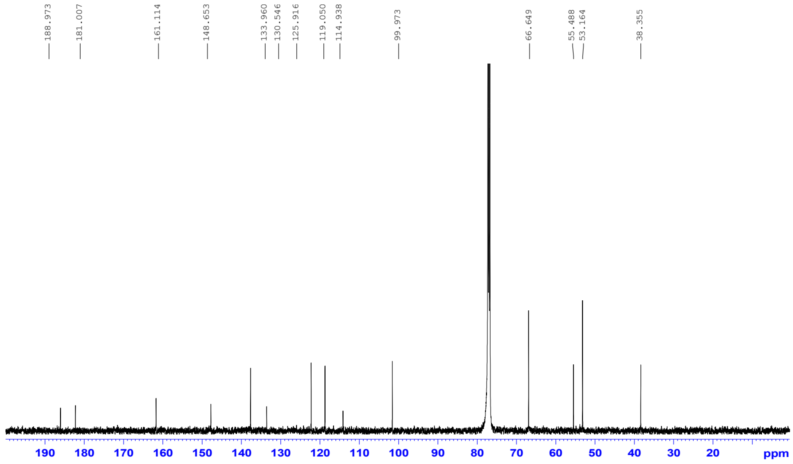

(**B**)

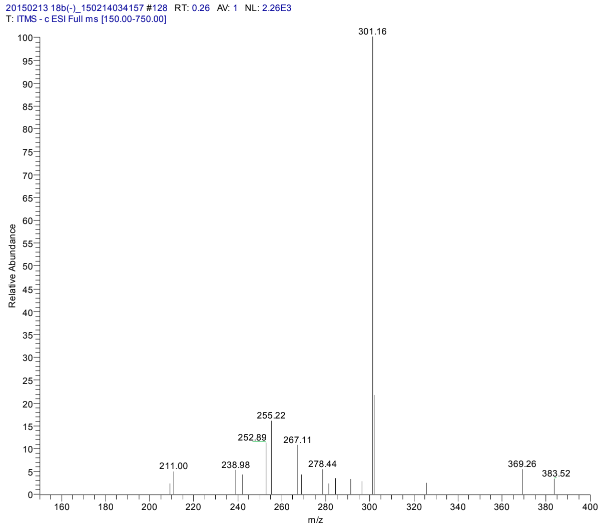

(**C**)

Figure S27. (**A**) ^{1}H-NMR spectra of compound **18b**; (**B**) ^{13}C-NMR spectra of compound **18b**; (**C**) LC-MS spectra of compound **18b**.

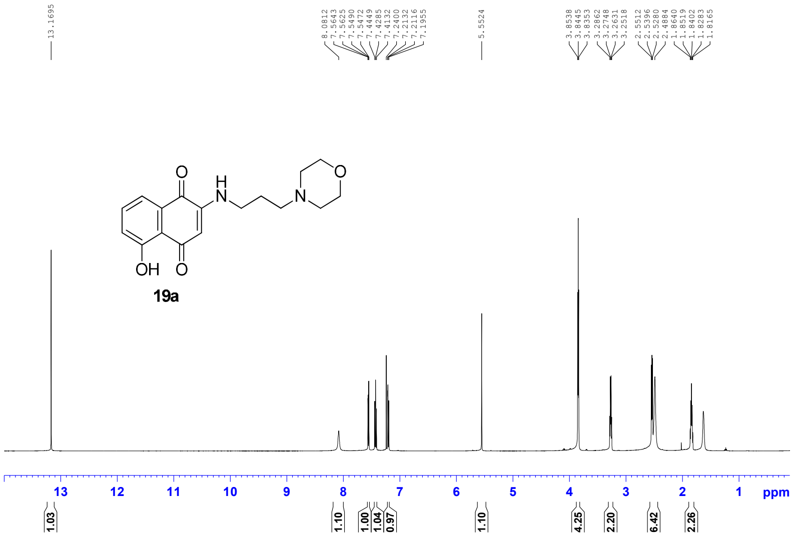

(A)

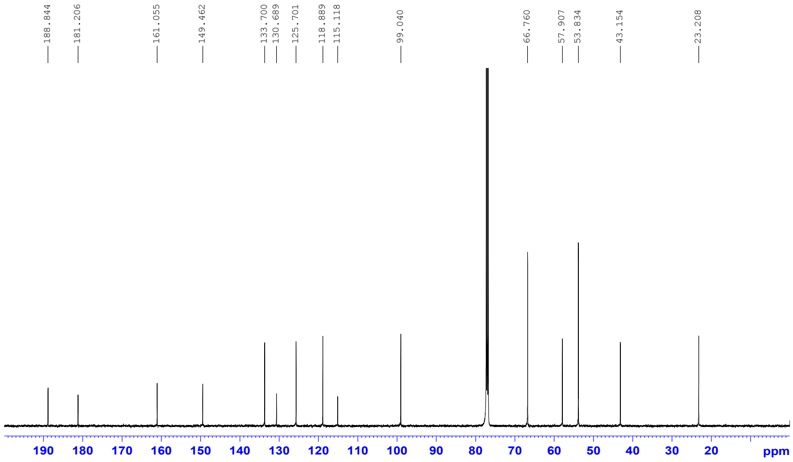

(B)

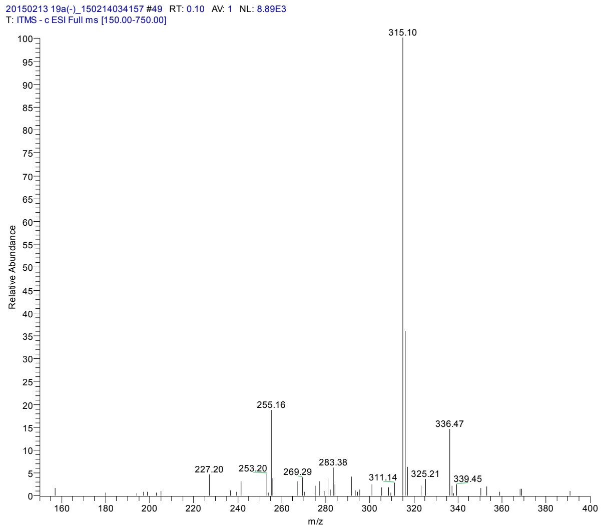

(C)

Figure S28. (**A**) ^{1}H-NMR spectra of compound **19a**; (**B**) ^{13}C-NMR spectra of compound **19a**; (**C**) LC-MS spectra of compound **19a**.

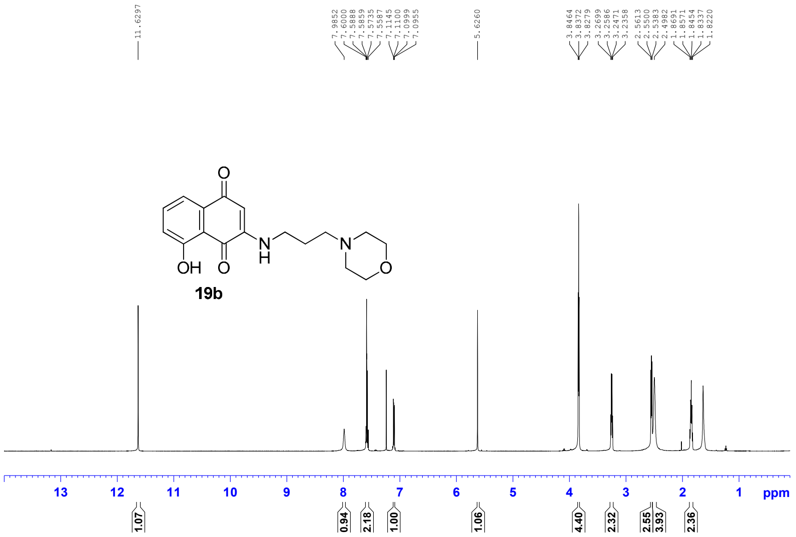

(A)

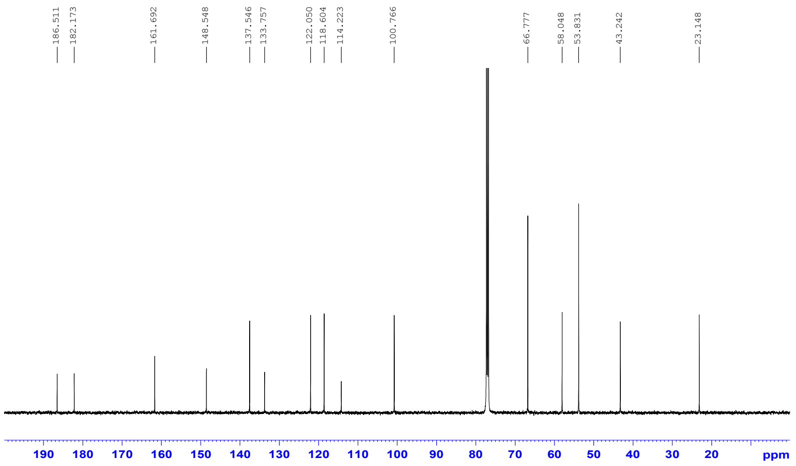

(B)

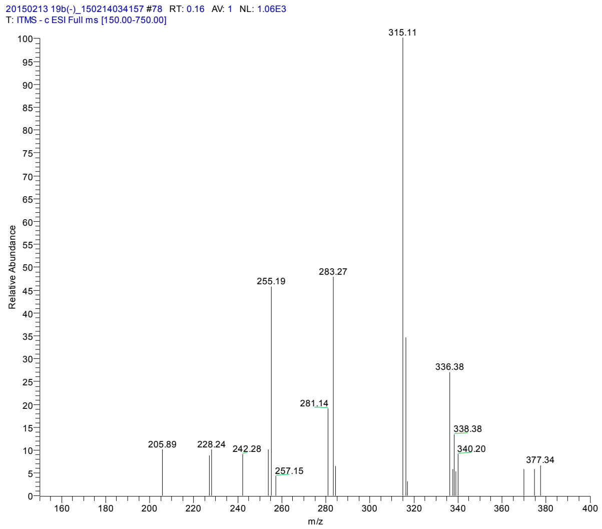

(C)

Figure S29. (**A**) ^{1}H-NMR spectra of compound **19b**; (**B**) ^{13}C-NMR spectra of compound **19b**; (**C**) LC-MS spectra of compound **19b**.
